# More Than a Service: Values of Rivers, Wetlands and Floodplains Are Informed by Both Function and Feeling

**DOI:** 10.1007/s00267-023-01900-2

**Published:** 2023-10-27

**Authors:** Cherie J. Campbell, Siwan Lovett, Samantha J. Capon, Ross M. Thompson, Fiona J. Dyer

**Affiliations:** 1 Centre for Applied Water Science, Institute for Applied Ecology, Faculty of Science and Technology, University of Canberra, Bruce, ACT 2601 Australia; 2 Australian River Restoration Centre, Canberra, ACT 2601 Australia; 3 Australian Rivers Institute, Griffith University, Nathan, QLD 4111 Australia

**Keywords:** Perspectives, Environmental flows, Riparian vegetation, Social-ecological systems, Storytelling, Values

## Abstract

How people value rivers, wetlands and floodplains influences their attitudes, beliefs and behaviours towards these ecosystems, and can shape policy and management interventions. Better understanding why people value rivers, wetlands and floodplains and their key ecosystem components, such as vegetation, helps to determine what factors underpin the social legitimacy required for effective management of these systems. This study sought to ascertain perspectives on the value of non-woody vegetation in river-floodplain systems via an online survey. The survey found that participants valued non-woody vegetation for their provision of a range of ecosystem functions and services, with strong emphasis on ecological aspects such as regulation functions, habitat provision and biodiversity. However, the inclusion of a question framed to focus on stories or narratives resulted in a different emphasis. Responses indicated that non-woody vegetation, and rivers, wetlands and floodplains were valued for the way they made people feel through lived experiences such as recreational activities, personal interactions with nature, educational and research experiences. This highlights the important role of storytelling in navigating complex natural resource management challenges and ascertaining a deeper understanding of values that moves beyond provision of function to feeling. Improved understanding of the diverse ways people value and interact with river-floodplain systems will help develop narratives and forms of engagement that foster shared understanding, empathy and collaboration. Appreciation of plural values such as the provision of functions and services along with the role of emotional connections and lived experience will likely increase lasting engagement of the general public with management to protect and restore river-floodplain systems.

## Introduction

The majority of the world’s rivers, wetlands and floodplains (RWFs) are under considerable stress because of human impacts (e.g., Best 2019). The challenge in achieving better management of freshwater ecosystems is that they occur at the food-energy-water nexus, with competing demands on water which play out across national boundaries (e.g., Cai et al. 2018; Keskinen and Varis 2016). This necessitates decision on trade-offs between uses that require social and political inputs enabled by informed community engagement (e.g., Leigh and Lee 2019; Nones 2016; Priscoli 2004). This is leading more researchers and managers to consider river basins as social-ecological systems (e.g., Gain et al. 2021; Godden and Ison 2019). At the heart of these systems is the need for an understanding of how freshwater ecosystems are valued by humans, and how those values may influence decisions in water policy and management (O’Donnell et al. 2019). There is increasing recognition of the influence of societal values and perceptions of nature on environmental beliefs and behaviour and the subsequent implementation or success of conservation and restoration efforts (Conallin et al. 2022; Galbraith et al. 2021; Ives and Kendal 2014; Smith et al. 2014; Steg et al. 2014).

As stated by Ives and Kendal (2014) “*every* [ecological management] *action and intervention is drenched in human values of some kind*”. How people value and interact with RWFs will influence the aspects of these places people wish to protect or restore (Martin and Czellar 2017; St John et al. 2010). For example, the need or desire to undertake particular activities, including spiritual ceremonies, household activities, or recreational pursuits such as fishing or swimming, can influence perceptions of desirable water depth, quality, velocity or macrophyte cover (Keeler et al. 2015; Lokgariwar et al. 2014; Sharma et al. 2020; Verhofstad and Bakker 2019). Biospheric (nature-centred) values emphasise the intrinsic worth of species and the environment (Steg and de Groot 2012) and, as such, influence committed action for nature and biodiversity (Fornara et al. 2020). Social-ecological systems such as RWFs have value in and of themselves (intrinsic values), value in terms of what they do or provide for people (instrumental values), as well as value through complex relationships and responsibilities (e.g., preferences, principles and virtues) that people have with these systems (relational values) (Chan et al. 2016; Chan et al. 2018).

RWFs are some of the most threatened and degraded ecosystems in the world (Bradshaw et al. 2021; Dudgeon 2019; Reid et al. 2019). The need to protect and restore these systems is well recognised globally (Arthington 2021; Tickner et al. 2020), with environmental flows or environmental water management (EWM) identified as a major priority (Anderson et al. 2019; Arthington et al. 2018; Maia 2017). EWM, does however, require social legitimacy for successful implementation (Doolan et al. 2017; Johnson et al. 2021; O’Donnell et al. 2019). Trust, transparency, a shared understanding and acceptance of the problem, a common vision of success, and building mutually respectful, ongoing relationships, have been highlighted as key to social legitimacy (Dare et al. 2014; Johnson et al. 2021; O’Donnell et al. 2019). Understanding why the outcomes of EWM matter, and to whom, is an important aspect of legitimacy and establishing a shared understanding and vision (O’Donnell et al. 2019). Better understanding values can help inform processes that foster shared understanding and just management of environmental water (Anderson et al. 2019; Gustafson et al. 2022; Ives and Kendal 2014; O’Donnell et al. 2019).

In river-floodplain systems vegetation comprises long-lived woody vegetation such as trees and large shrubs, along with a diverse array of non-woody vegetation (NWV) such as floating plants, submerged macrophytes, herbs, grasses, sedges and rushes, sub-shrubs and tall reeds. Vegetation plays a critical role in river-floodplain ecosystems (Capon et al. 2016; Riis et al. 2020), is often a focus of EWM (Cogle et al. 2010; Colloff et al. 2015; Shafroth et al. 2017) and is a visually dominant component of the aesthetics of RWFs. This paper is part of a body of research specifically focused on NWV in RWFs. This body of research is interested in the notion of ‘what is good’ and rethinking how the construct of condition is used to envisage and evaluate NWV outcomes to EWM. This includes better understanding the functions and values provided by NWV (see Campbell et al. 2021; Campbell et al. 2022), to *“improve our knowledge on the social dimensions of riparian* [including wetland and floodplain] *vegetation”*, which is one of the top ten challenges for riparian vegetation science and management recently identified by global experts (Rodríguez-González et al. 2022). This study sought to ascertain perspectives on the value of NWV in RWFs using an online survey instrument and contribute to building knowledge on the social dimensions of vegetation in RWFs.

## Methods

### Survey design and distribution

We designed and implemented an online survey to ascertain perspectives about the value and function of NWV in river-floodplain systems (Online Resource 1). This paper describes the outcomes of a sub-set of the broader survey, focussing on survey questions that addressed the research question: what is the value of NWV in RWFs? (Table 1).

**Table 1:** Details of, and relationship between, research question, survey questions, question format, options provided, and data analysis approach

Research question	Applicable survey questions	Question type	Analysis approach
*What is the value of NWV in rivers, wetlands and floodplains?*	*Q2.1 Do you value NWV?*	Closed-format multiple choice 6-point Likert scale: 0 = ‘I don’t know’, 1 = ‘not at all’, 2, 3 = ‘moderately’, 4, 5 = ‘very highly’ (1 selection allowed)	The number and proportion of responses was calculated for each option provided
	If the response to Q2.1 was 1 or 2: *Please expand on why you do not value NWV?*	Open-ended text responses	Common themes were identified to define nodes. Text responses were then coded against defined nodes in NVivo 12 Pro. The number of coded entries against each node was graphed for each question.
	If the response to Q2.1 was 3 or higher: *For what broad reasons do you value NWV?*	Open-ended text responses	
	*Please share any stories, thoughts, or memories about rivers, wetlands and floodplains that illustrate their value and importance to you*	Open-ended text responses	
Contextual information regarding survey participants	*Q1.1 Please select ALL options that describe your interest in rivers, wetlands, floodplains and environmental water management*	Closed-format multiple choice 10 options provided: (i) amateur naturalist/environmentalist, (ii) currently reside (or have previously lived) in a river-floodplain community, (iii) irrigator/farmer, (iv) professional – current or previous employment in environmental water management or research, (v) professional – current or previous employment related to rivers, wetlands or floodplains, (vi) recreational, (vii) student – study/research related to rivers, wetlands, floodplains or environmental water management, (viii) traditional custodian, (ix) other, (x) not interested in rivers, wetlands, floodplains or environmental water management (multiple selections allowed)	The number and proportion of responses was calculated for each option provided
	*Q1.2 Please select ONE option that describes your interest in rivers, wetlands, floodplains and environmental water management*	Closed-format multiple choice Same 10 options provided as for Q1.1 above but with only one selection allowed	
	*Q1.3 How would you describe your level of knowledge in relation to NWV and environmental water management?*	Closed-format multiple choice 5-point Likert scale: 1 = ‘no knowledge’, 2 = ‘limited level of knowledge’, 3 = ‘moderate level of knowledge’, 4 = ‘high level of knowledge’, 5 = ‘expert’ (1 selection allowed)	

Survey questions comprised a combination of closed-format items (i.e., multiple choice) and open-format text entry. Closed-format (standardised) questions have the benefit of ensuring all respondents consider the same options, and data can be analysed using quantitative statistical techniques (Toepoel 2016; Wolf et al. 2016). Open-format questions elicit unconstrained views and can be analysed using qualitative data analysis software (Toepoel 2016; Wolf et al. 2016).

We used the survey software Qualtrics (Qualtrics, Provo, UT) to host the survey. An anonymous survey link was created and distributed to known professional contacts, through academic and professional networks, including university affiliations, the Flow-MER program, the Australian River Restoration Centre, and the Australian Freshwater Sciences Society. It was also promoted through a webpage and social media (i.e., Facebook and Twitter). Recipients were also encouraged to distribute the survey through their own networks. All authors are based in Australia with experience and networks across the Murray-Darling Basin (MDB) (and other regions). While the study was not designed to elicit place-based values we acknowledge the study has an Australian and MDB bias. The survey was available for nine weeks, opening in early March 2021 and closing mid-May.

In total, 165 surveys were completed. Based on their responses, survey participants were guided through a subset of questions as they related to their area of expertise and interest. The number of participants who responded to individual questions is available in Online Resource 2.

### Survey response processing and analysis

Survey responses were processed in Qualtrics and exported to Microsoft Excel. Closed-format questions were graphed in Microsoft Excel to assess the proportion of responses to multiple choice categories. Responses to open-format text questions were analysed in NVivo 12 Pro (QSR International Pty Ltd). In line with recognised qualitative research approaches (e.g., de Casterle et al. 2021; Elo and Kyngaes 2008) text responses were read and reread to identify common themes prior to the systematic coding of responses against identified nodes in NVivo 12 Pro. Common themes broadly aligned with recognised categories in literature concerning ecosystem functions and services (e.g., Capon et al. 2013; de Groot et al. 2002), which are seen as a structured way to link the functioning of ecosystems to human benefits and values derived from those ecosystems (de Groot et al. 2002). Identified themes were aligned with broad categories of ecosystem functions and services, and guidelines for assigning text responses against the identified nodes were developed to enable consistent interpretation of responses (see Online Resource 2). The broad categories of ecosystem functions and values were: (i) regulation functions, such as water regulation or soil retention, (ii) habitat functions, such as the provision of habitat to live, forage, breed etc., (iii) biodiversity functions, which included references to intrinsic value, (iv) production functions, which specific to the context of this study referred to activities of commercial value, and (v) information functions, which included wellbeing-emotional connections, aesthetic, recreational, educational and cultural values (see Online Resource 2 for more information). The use of the term cultural values in this study, and in the context of EWM in Australia, is linked to Australia’s First Nations Peoples and Aboriginal water rights (e.g., Moggridge and Thompson 2023; Moggridge et al. 2019; Moggridge and Thompson 2021), rather than the broader use of the term cultural values or cultural ecosystem services (CES) as applied in other literature (e.g., de Groot et al. 2005; Fish et al. 2016b; Millennium Ecosystem Assessment 2005). Information functions, and the components considered under this category, should be viewed as analogous with the broader CES concept. Text responses, or portions of responses, were coded against the identified nodes and graphed to display the proportion of coded entries against each node for different questions. Quotes used throughout this paper may be portions of a full response; full responses from individual participants to open-ended survey questions are available in Online Resource 2. Table 1 displays applicable survey questions, question type, options provided, and the data analysis approach.

## Results

### Participant knowledge and relationships with RWFs

**Fig. 1:**
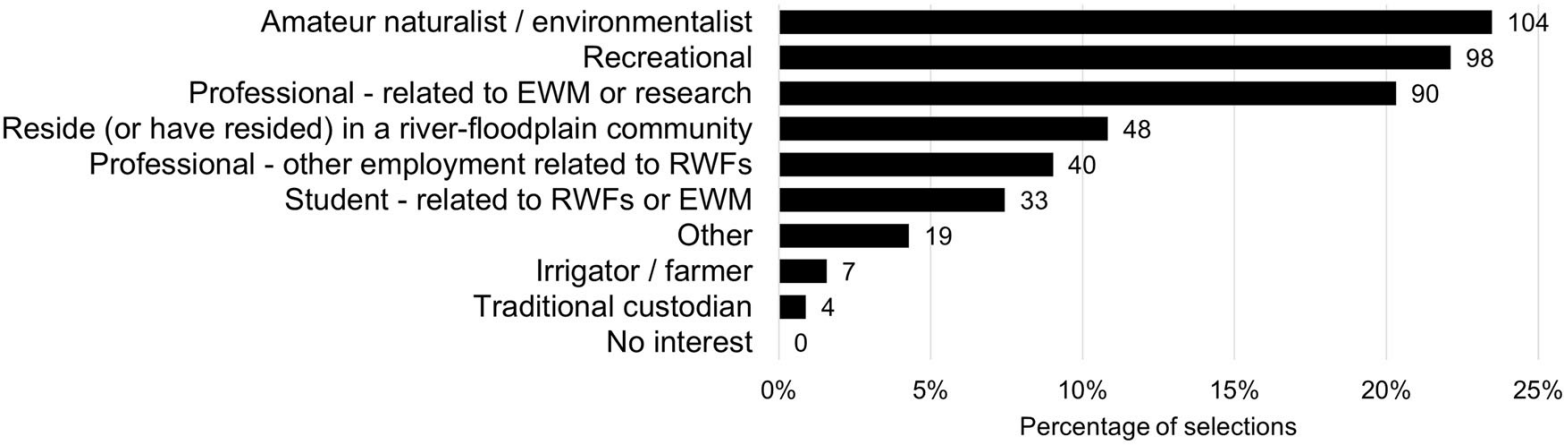
Number and percentage of selections in each of the options provided for interests or relationships with rivers, wetlands, floodplains and environmental water management, *n* = 443 selections from 165 respondents, EWM environmental water management, RWFs rivers, wetlands, and floodplains. Note that each respondent was able to select multiple options

Seventy-two percent of survey participants self-identified as being at least moderately knowledgeable in relation to NWV and EWM, with 16% indicating an expert level of knowledge, and less than 3% indicating they had no knowledge of NWV and EWM (Online Resource 2). Survey participants covered a range of interests in RWFs. The three most common interests or relationships were amateur naturalist/environmentalist (23%), recreational (22%) and professional involvement related to EWM or research (20%) (Fig. 1). More than 70% of respondents identified with more than one of the 10 types of interests or relationships provided (Online Resource 2).

Additional text responses provided by participants focusing on individual interests or relationships with RWFs included artistic pursuits (e.g., *“photography”*, *“artist of Australian plants and habitats with interest in different ecosystems”*), volunteer roles (e.g., *“Landcare volunteer – mostly regeneration on creeks and rivers locally”*, *“volunteer with wetland care group and Waterwatch citizen science programme”*), and landholders that didn’t fit the irrigator/farmer category (e.g., *“landowner of conservation covenant areas working with our neighbour to re-establish our riparian areas as healthy, complex and functional wetland habitats”, “landholder with creek frontage (not irrigator or farmer)”*). Additionally, the *“intrinsic value of the environment”* itself was mentioned along with deeply personal connections to RWFs, such as:

“*Yorta Yorta Country: The way in which I connect, see, feel, hear, taste, sound, connect (sing, language) and speak of Country is essential to the way in which the water must be there for the rivers, wetlands, floodplain and biodiversity. If it is no longer there, then I will become ill and upset. Seeing Country from the sky brings sadness. There is not much left*”,

“*Essential to environment and hence my own survival*” and

“*I want to say LOVE and NEED (as in dependence for life) which I imagine feeling when I am present in a wetland and don’t seem adequately represented by the categories of amateur naturalist/environmentalist or recreation*”.

### What are the values of NWV and of RWFs?

#### Do you value NWV? Why, or why not?

The majority of respondents (71%) value NWV very highly (i.e., a score of 5 out of 5), with virtually all respondents (99.4%) indicating a moderate or greater value for NWV (i.e., a score of 3, 4 or 5 out of 5), with only a single participant responding with ‘I don’t know’ (Online Resource 2).

One hundred and thirty-nine survey participants provided text responses to explain the reasons they value NWV. From the 139 responses, 373 individually coded values were identified, with an average of approximately three values identified per response with a range from 1 to 10.

**Fig. 2:**
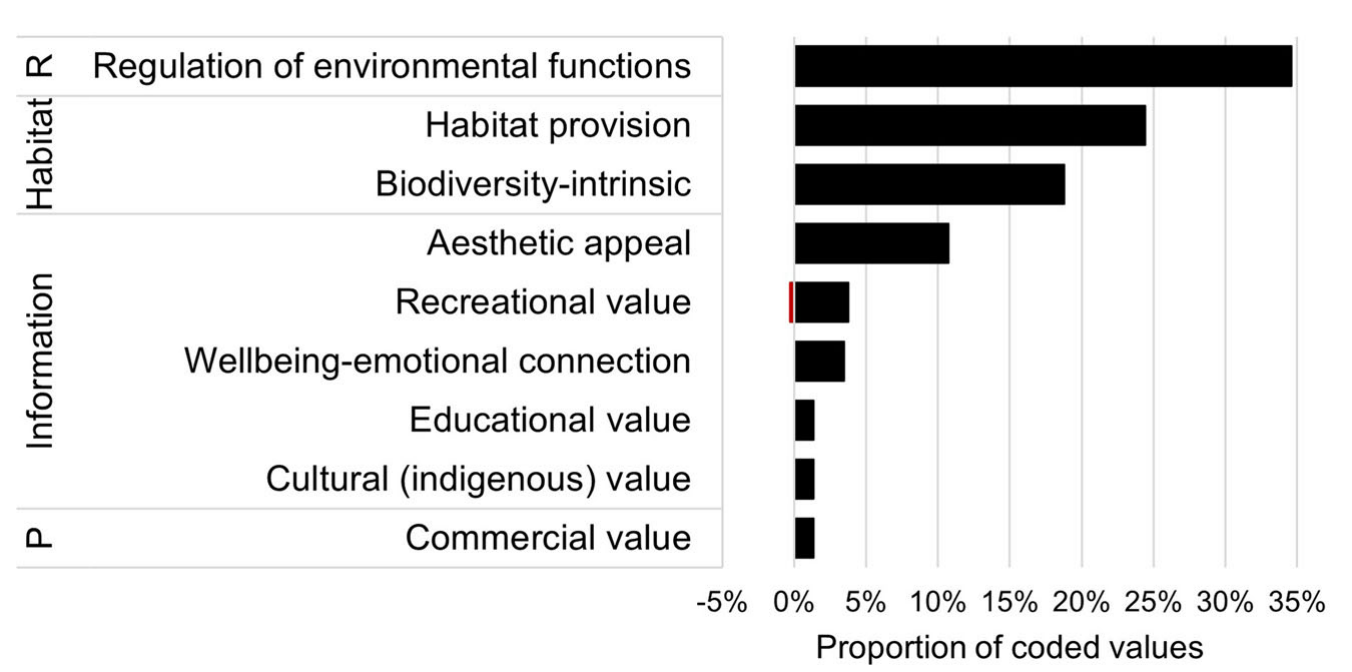
Proportion of values identified in response to the question ‘for what broad reasons do you values NWV’ that align with identified themes and broad categories of ecosystem functions and services; R regulation, P production. Red bars represent values (*n* = 1) that were explicitly stated as negative by the respondent. *n* = 373 coded values from 139 responses. Note ‘Information’ is analogous with cultural ecosystem services

Seventy-eight percent of identified values related to ecologically-focused values, such as the regulation of environmental functions (35%), habitat provision (24%), and biodiversity-intrinsic values (19%) (e.g., *“It includes a lot of plant communities that have value in their own right”*) (Fig. 2). Habitat provision included habitat for breeding and juveniles (e.g., *“nesting grounds for birds”*, *“this vegetation provides habitat for the juvenile stages of fish and crayfish”*), foraging and feeding (e.g., *“food sources (bees, insects)”*, *“foraging habitat for waterbirds such as Australian bitterns and ibis”*), and habitat corridors for connectivity (e.g., *“habitat for migratory birds”*).

Other identified values related to aesthetic appeal (11%) (*“they’re just beautiful to look at!”*), recreational value (4%), wellbeing or emotional connection (3%) (*“Appreciating these aspects of nature makes my heart sing!!”*), educational (1%) (e.g., *“measuring tool for the health of the water system”*), and cultural values (1%) (e.g., *“as a Wiradjuri supermarket/classroom”*). The remaining 1% related to commercial value (e.g., *“renewal of river systems and agricultural lands if well managed”*, *“Farmer’s daughter and amateur conservationist appreciating the value of the wetland for agricultural purposes and…”*).

A range of recreational activities were mentioned including walking or hiking, birdwatching, camping, canoeing, and fishing. The recreational value category contained one of only two mentions of negatively perceived values of NWV: *“sometimes* [NWV] *get in the way of recreation”* along with the more generic comment “*it* [NWV] *can* [be] *a benefit and pest”*.

**Fig. 3:**
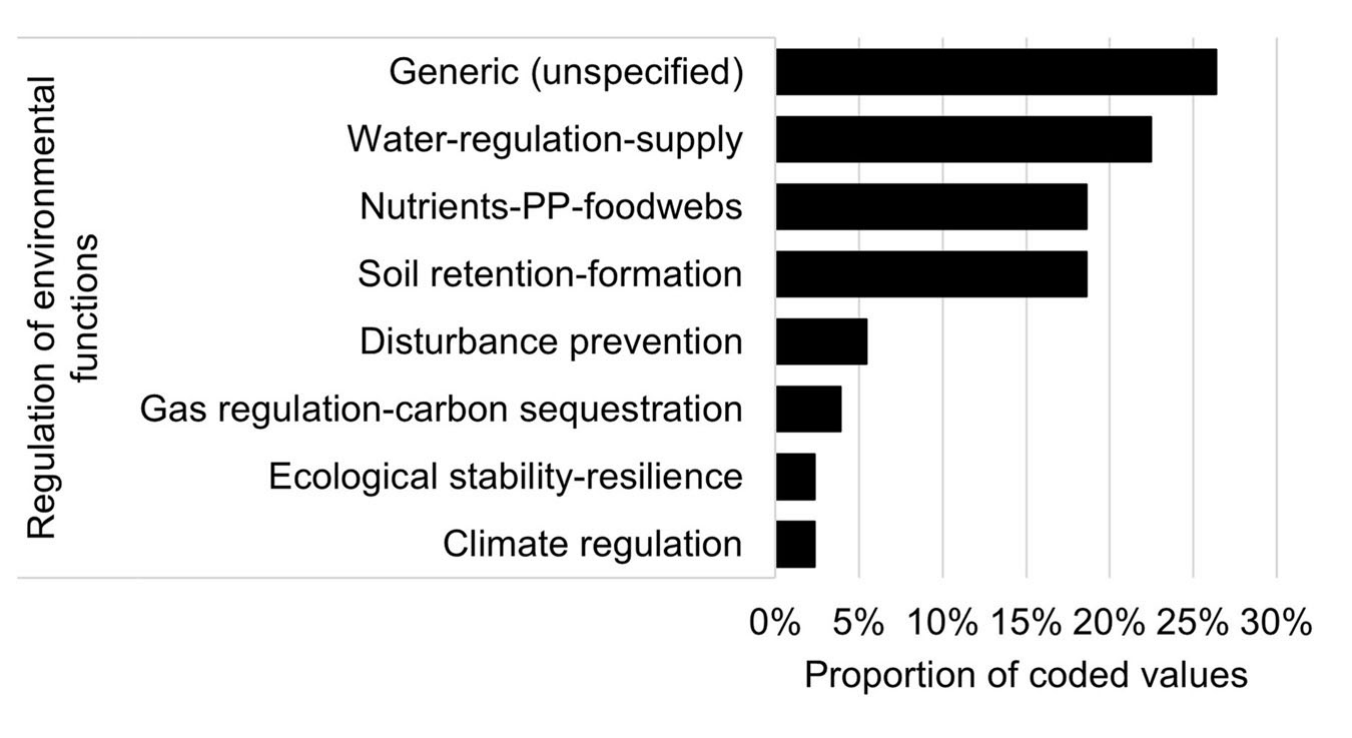
Proportion of values that align with categories within ‘regulation of environmental functions’ (based on de Groot et al. (2002); see also Online Resource 2). *n* = 129 coded values from 139 responses (PP primary production)

Values coded against the regulation of environmental functions theme (35% in Fig. 2) were further assessed to identify specific regulation functions (Fig. 3). More than 25% of these regulation functions were broadly described in the text responses and could not be further refined (e.g., *“services and functions NWV provides”*). Where specific regulation functions could be determined, these related to water regulation and supply (22%) (e.g., *“importance of filtering nutrient, sediment, and contaminant inputs within floodplain systems”*, *“it* [NWV] *plays important roles in water supply and quality”*), nutrient regulation, primary production and food web processes (19%) (e.g., *“exchange or redistribution of energy and nutrients”*), soil retention and formation (19%) (e.g., *“bank and soil stabilisation”, “protection from erosion”*), disturbance protection (5%) (e.g., *“flood buffer zone”*), gas regulation and carbon sequestration (4%) (e.g., *“respiration and carbon moderation”, “carbon sequestration”*), ecological stability and resilience (2%) (e.g., *“ecosystem resilience”*) and climate regulation (2%) (e.g., *“regulates temperature”*) (Fig. 3).

#### Stories, thoughts, and memories about RWFs

Eighty respondents shared stories, thoughts or memories about RWFs that illustrate their value and importance. These text responses were assessed against the same themes as shown in Fig. 2.

**Fig. 4:**
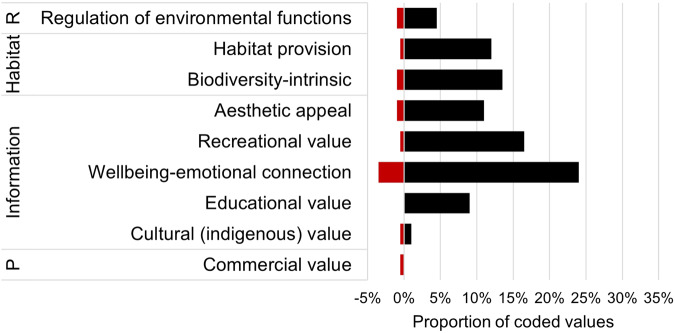
Proportion of values identified in response to the question ‘please share any stories, thoughts, or memories about rivers, wetlands and floodplains that illustrate their value and importance to you’ that align with identified themes and broad categories of ecosystem functions and services; R regulation, P production. Red bars represent values that were expressed by respondents as negative contrasts, negatively altered processes, or negative consequences. *n* = 202 coded values from 80 responses. Note ‘Information’ is analogous with cultural ecosystem services

Sixty-eight percent of values identified were associated with the broad ecosystem function category of ‘information functions’ such as wellbeing-emotional connection (28%), recreational value (17%), aesthetic appeal (12%), educational (9%) and cultural values (2%) (Fig. 4). Responses coded under wellbeing-emotional connection used language such as *rejuvenating, relaxing, exciting, peaceful, calm, love, joy, treasured, remarkable, surprising, immersed in nature, an absolute delight, so special, privileged* [to have been there], with memories that were *lovely, strong, fondest* and *wonderful*. Recreational activities were varied and included birdwatching, camping, swimming, kayaking/canoeing/boating, catching fish/yabbies/crayfish, duck-hunting, walking, and photography. Aesthetics – sights and sounds – were valued in their own right (e.g., *“The loveliness of a native buttercup field in clear shallow floodwater under the filtered light of magnificent river red gums”*) or in relation to other activities (e.g., *“…the aesthetics provided by NWV has usually been a significant factor in that choice* [of camping location]*”*). Educational experiences occurred through school and university fieldtrips, work and volunteer opportunities, and via landholder restoration projects (e.g., *“Our revegetation projects have highlighted to me the complexity of these systems and how important it is to protect the variety of wetlands we have in Australia. It has allowed me to learn much more about the complexity of those ecosystems.”*). Interspersed within the ‘information functions’ were numerous responses that highlighted relational values and links to childhood or family, for example:

“*I loved spending time exploring wetlands as a child, just discovering all the plants and creatures that lived in them. I used to make little reed baskets and collect tadpoles and invertebrate larvae to watch and rear. Favourite species are dragonflies, damselflies and frogs, plus Eleocharis and Nardoo*”.

*“Taking my son to explore along the Cotter River, sharing my knowledge of which plants are native and which not.”*

Thirty-four percent of values identified related to ecologically-focused values, such as biodiversity-intrinsic values (15%) (e.g., *“The whole ecosystem is very rich and unique.”*), habitat provision (23%), and the regulation of environmental functions (6%) (Fig. 4). Habitat was mentioned in terms of a wide variety of birds, including for bird breeding and foraging, along with frogs, invertebrates, and fish. Regulation functions included temperature control (e.g., *“*[riparian habitats are] *cool compared with the surrounding cropland”*), increased rates of coastal land-loss from the loss of saltmarsh wetlands, and water filtration (e.g., *“…I love the fact that* [an urban wetland] *is able to be swum in because of the work that has gone into making flood catchment reservoirs with natural filters (vegetation)”*).

## Discussion

This study sought to ascertain perspectives on the value of NWV in RWFs using an online survey instrument. The two questions we posed yielded responses with differing thematic emphases. The first question elicited perspectives with an emphasis on ecological functions provided, while the second elicited perspectives with an emphasis on feelings and experiential interactions. We believe the two distinct classes of responses is highly informative in terms of social engagement around water management. There are many different ways to consider values and their influence on environmental management. Ives and Kendal (2014) highlight the distinction between underlying values (i.e., values which shape people’s perception of the world e.g., biocentric, social-altruistic, hedonic, egoistic) and assigned values (i.e., the values that people assign to things in the world or their relative worth e.g., monetary value or the value of goods and services provided). Chan and others (Chan et al. 2016; Chan et al. 2018) advocate values of nature in and of itself (intrinsic values), in terms of what nature does or provides for people (instrumental values), as well as considering the complex relationships and responsibilities (e.g., preferences, principles and virtues) that people have with nature (relational values). Instinctively, the provision of ecosystem functions and services for a human benefit aligns more with the idea of assigned or instrumental values than underlying or intrinsic values. Though different types of values are undoubtably related and deeply intertwined (Chan et al. 2018; Ives and Kendal 2014), with growing recognition of the need to incorporate relational values into the framing of ecosystem services (e.g., Fish et al. 2016b). The benefit provided by an ecosystem function can be assigned a value, but this will be influenced by the way that individual perceives or feels the world. For example, valuing the intrinsic right for species to exist, or the benefit to fauna through habitat provision, may be fostered by a biocentric value orientation. Valuing the benefit to humans from water filtration may be influenced by a social-altruistic value-orientation, while the pleasure derived from viewing an attractive landscape may be influenced by a hedonic value orientation. The above examples focus on the interplay between assigned and underlying values, though Chan et al. (2018) highlight the interplay between values can be far from binary. In environmental management there is a need to understand different types of values (e.g., Chan et al. 2018; Ives and Kendal 2014) with a recognition that different methodological approaches are likely to draw out different values.

The framing of the two questions asked in the survey is likely to have had an influence on the types of values mentioned, the way they were expressed in text responses, and the interpretation of the values provided by NWV and RWFs more broadly. The wording of the first question *‘for what broad reasons do you value NWV’* appears to have triggered a thought process regarding the more tangible usefulness or provision of services by NWV – the known functions of NWV. The second question asked specifically for stories, thoughts or memories which is likely to have prompted emotive language and a sense of feeling that can be harder to categorise in terms of ecosystem functions and services (though readers are directed to literature regarding CES such as Fish et al. (2016a); Pastor et al. (2022)). We acknowledge that many of the experiential interactions and relational values highlighted in the storytelling question are likely to occur, and be remembered, because of underlying ecological processes that support the flora, fauna and ecological conditions that contribute to the quality of the interaction (e.g., “*Another strong memory I have is walking along a boardwalk at a wetland with my father. The two of us stopping and waiting quietly for a long time, then excitedly pointing out a platypus that emerged. It was so special*.”). The process of asking for ‘*stories, thoughts, or memories about RWFs that illustrate their value and importance to you’* appears to have evoked a stronger emphasis on experiential interactions, emotions and wellbeing – how NWV and RWFs make people feel. By including this question we have a greater appreciation of the feelings that underpin survey participants’ attitudes and beliefs towards NWV and RWFs.

The contrasting sets of values reflected in responses to the two questions illustrates the benefit of ascertaining values in multiple ways (e.g., Pastor et al. 2022) and supports a growing interest in the use of storytelling and narratives. Vigliano Relva and Jung (2021) illustrate how narratives can help develop a richer understanding of social-ecological conflicts by using marine fisheries management as an example. They argue that storytelling can help to unravel the values and beliefs that shape different narratives to give a greater understanding about what drives conflicts (Vigliano Relva and Jung 2021). Developing this understanding increases the chance of finding mutually beneficial solutions (Vigliano Relva and Jung 2021). Similarly, Liguori et al. (2021) used storytelling *“to imagine, interrogate and plan for a future that communities might collectively wish to subscribe or adapt to”* as part of decision making around drought risk in a catchment in the United Kingdom. The Liguori et al. (2021) example is about finding novel solutions to realistic drought scenarios to help build both human and ecological resilience.

By accepting that river-floodplain systems are social-ecological systems we accept that the problems, benefits and solutions associated with these systems are social and ecological. Therefore, the need to better understand values should be tackled via a range of approaches, including storytelling and narratives, that recognise both ecological and social dimensions. For example, considering the intrinsic value of systems, their provision of functions, along with relational experiences and feelings.

### Caveats and future considerations

#### Representativeness of perspectives

While the research aimed to ascertain broad perspectives on the values of NWV and RWFs, the respondents were highly engaged with water and river management (Fig. 1), and this study is unlikely to have captured all community perspectives. There are three notable perspectives that are underrepresented or lacking: traditional owners, farmers/irrigators, and the section of the community that are apathetic, disinterested or hostile to EWM. The introduction to the survey (available in Online Resource 1 and web access) was specifically seeking participants with a love of RWFs to better understand the components of NWV (and RWFs more broadly) that help to create those connections. As evidenced in Fig. 1, this led to a cohort of participants who all had at least some interest (likely positive) in RWFs or EWM. This survey was not seeking to understand why people do not engage with RWFs or EWM. Seven participants identified a relationship to RWFs as irrigator/farmer, with an additional two participants identifying (via other) as landowners but not irrigators or farmers (see Online Resource 2). While less represented than other relationships in this study, nine perspectives from individuals who identify as landholders, irrigators or farmers is in line with other surveys (e.g., Allan and Watts 2022). We acknowledge targeted, in person survey approaches may be required to gain additional perspectives from landowners, farmers or irrigators, such as undertaken by Allan and Watts (2022); or Doehring et al. (2023). Four participants identified a relationship to RWFs as traditional custodians. Different, culturally appropriate, engagement methods are required to incorporate broader aboriginal perspectives on the values and functions of NWV (Douglas et al. 2019; Moggridge et al. 2019). Culturally appropriate engagement methods are likely to include face-to-face discussions undertaken on country. These discussions can only be entered into with the consent of local elders and communities after establishing relationships of trust and respect, along with clear agreements regarding the involvement of traditional owners and the use of cultural intellectual property. Studies seeking to understand the values of traditional owners should be designed and led by traditional owners for the benefit of traditional owners (e.g., Moggridge et al. 2019). Additionally, online survey formats may limit access to certain demographics or sectors of the community and, as such, may be difficult to generalise to a broader population (Evans and Mathur 2018). While we acknowledge certain community perspectives may be underrepresented this in no way diminishes the insights and experiences from individuals nor the knowledge that is gained by considering thoughtful and often in-depth responses from individuals – the value of *n* = 1 lived experience (see Sandelowski 1995, 1996). Readers are strongly encouraged to read the original responses available in Online Resource 2.

The survey was not designed to reflect the values of a specific spatial location but rather to reflect the values individuals associated with NWV and RWFs more broadly. We acknowledge however that the survey was distributed via known contacts and professional networks and therefore, based on the authors’ affiliations, the values and perceptions presented in this study are likely to represent a cross-section of the community (noting the exceptions above) that are relatively engaged and well-informed regarding EWM in the MDB in Australia. Based on specific locations mentioned in text responses (Online Resource 2), participants have drawn on experiences that cover the MDB in Australia, including the northern basin, headwater catchments, southern basin and lower lakes. Experiences, however, also encompass catchments outside the MDB covering south-eastern Australia, Queensland, Tasmania, Northern Territory and Western Australia, with one response specifically mentioning river systems outside of Australia.

#### Alignment of themes with ecosystem functions and services and the framing of values

Identifying themes and aligning them with broad categories of ecosystem functions and services aims to quantify the functions and values represented in open-ended text responses. The approach is, however, woefully inadequate at conveying the poetry and emotion expressed in many of the responses, particularly to the question asking for stories, thoughts and memories. Readers are strongly encouraged to read the original responses available in Online Resource 2 to gain a deeper appreciation of the richness of answers to this question.

The theme ‘wellbeing or emotional connection’ was identified as part of the process of reading and analysing text responses to determine key nodes for further assessment. In terms of aligning our themes with categories of ecosystem functions and services we largely followed those in Capon et al. (2013); and de Groot et al. (2002). While these framings of ecosystem functions and services recognise ‘information’ functions such as ‘aesthetic information’, ‘cultural and artistic information’ and ‘spiritual and historic information’, we included ‘wellbeing and emotional connection’ to explicitly recognise values that focused on the way NWV, and RWFs more broadly, made people feel (e.g., surprise, amazement, joy, peace). For example, *“Appreciating these aspects of nature makes my heart sing!!”*, and *“capacity to surprise (by presence; by recovery; by beauty)”*. We also wanted to capture the link that was sometimes expressed between feelings and wellbeing, such as the following response:

“*A place to wander or sit to ponder on my day, reconnect with nature and the land around me. It helps me put things in perspective. Looking at how the plants change each day, month, season. The way the insects go about their business between the plants and some dipping onto the water top. Gazing at the reflections on the water and watching the plants wave in the breeze. It centres me. Helps me to manage my low mental health and feel gratitude. Like no matter what is going on in my life, this place will be here, doing its thing, each and every day*.”

We recognise that recent framings and reviews of RWF ecosystem services (e.g., Hanna et al. 2018; Petsch et al. 2023; Riis et al. 2020; Xu et al. 2020) incorporate a wider range of information or cultural services than Capon et al. (2013); or de Groot et al. (2002). For example, in Riis et al. (2020) cultural services (equivalent to the information section referred to in Capon et al. (2013); and de Groot et al. (2002)) includes the provision of experiential and physical interactions, as well as the association of mental or moral wellbeing with that and other ecosystem services such as sacred or religious values, existence, and bequest (Riis et al. 2020). However, while there is increasing recognition of a wider range of information or cultural services in the ecosystem services literature, they tend to be underrepresented or poorly integrated (e.g., Hanna et al. 2018; Xu et al. 2020) in assessments of value or importance, certainly in relation to floodplain-wetland vegetation (Riis et al. 2020; Rodríguez-González et al. 2022). Riis et al. (2020), in their review of ecosystem services provided by riparian [river-floodplain] vegetation, *“did not assign relative importance* [to CES] *because of a lack of data to support such assessment.”* The need to improve knowledge on the social dimensions of riparian vegetation is recognised as one of the top 10 challenges by a global collaboration of experts in this field (Rodríguez-González et al. 2022), along with the need to integrate social dimensions to develop a resilient and sustainable relationship between societies and river-floodplain ecosystems (Dufour et al. 2019).

In addition, values are a wide and contended concept that span multiple disciplines with different possible framings. Beyond the ecosystem functions and services discussed above, this paper was shaped by Ives and Kendal (2014) and their exploration of the role of values in ecological management. We appreciate there are other framings of values that could have been applied to this study (e.g., Chan et al. 2018; Fish et al. 2016b). As stated by experts in the field of values, (Chan et al. 2018) *“in some [] contexts, there is likely no need to distinguish these different conceptions of values – what matters is that there is a space to express what matters to people on their own terms.”* Regardless of the particular framing of values or ecosystem functions and services applied to this study, key outcomes include: (i) allowing the space for people to express why NWV and RWFs matter to them, (ii) recognising that there are multiple ways to view NWV values that cover their intrinsic worth, the functions they provide to humans and ecosystems, as well as complex relationships that cover emotive connections and feelings, and (iii) highlighting the role different approaches, such as storytelling or narratives, can play in drawing out different types of values.

#### The ‘value-action gap’

In this paper we assert that values influence attitudes, beliefs, and behaviours (Ives and Kendal 2014; Steg et al. 2014; e.g., Steg and de Groot 2012), which, in relation to natural resource management, can shape policy, management interventions and perceived outcomes (Galbraith et al. 2021; Smith et al. 2014; e.g., St John et al. 2010). While values are the focus of this study, we recognise that additional elements influence the ‘value-action gap’ (e.g., Blake 1999; Huddart et al. 2009) and affect, for example, transitions from values to behavioural-intent to action (Kulin and Seva 2021; Schirmer and Dyer 2018; e.g., Steg et al. 2014). Outcomes in the above citations highlight that the ‘value-action gap’ is influenced by a range of elements. Multiple frameworks have been proposed to help identified factors contributing to the value-action gap such as the IFEP framework (values, situational cues, goals) (Steg et al. 2014) and the VAIL framework (values, awareness, identity, lifestyle), the latter in the context of water-sensitive urban design (Schirmer and Dyer 2018). Factors contributing to the value-action gap can be individual, household or societal (Blake 1999; Huddart et al. 2009) through to the quality of national governments (Kulin and Seva 2021). We recommend future studies explore the ‘value-action gap’ in relation to EWM.

#### Reflection and collective learning

This study set out to ascertain perspectives on the value of NWV in river-floodplain systems, with the lead author approaching this research as a vegetation ecologist with an appreciation of ecosystem functions and services. The process of undertaking the research, however, has led to a greater appreciation of the diversity of values associated with NWV and RWFs and increased awareness of the role of values in EWM. This process and self-reflection will in turn influence future research and management undertaken by the authors. Anderson et al. (2019) advocate placing *“the acceptance that there are many different ways of seeing and knowing rivers at the core of environmental flow assessments”*. That acceptance needs to permeate through the values of those undertaking environmental flow assessments and translate into behaviour and actions. Vigliano Relva and Jung (2021) highlight that *“most current social-ecological conflicts are characterised by having multiple contested narratives about issues that stem from differences in perception, values and even different “reals”*. The role of reflection and collective learning is critical to accepting different narratives and working towards mutually beneficial outcomes (Allan and Watts 2022; Liguori et al. 2021; Vigliano Relva and Jung 2021).

#### Implications for EWM – what is the role of storytelling and narratives?

EWM is a human endeavour and, as such, “*every* [ecological management] *action and intervention is drenched in human values of some kind*” (Ives and Kendal 2014). We believe this study adds to the growing body of work highlighting the important role of storytelling in navigating complex natural resource management challenges, such as marine fisheries management (Vigliano Relva and Jung 2021), drought risk management (Liguori et al. 2021) and river restoration (Doehring et al. 2023). While it is outside the scope of this study to explicitly set out how storytelling and narratives could be used in EWM, we offer some insights into their potential role and encourage further research and discourse into this area. Storytelling and narratives are one approach to gathering or communicating data and information, for working through conflicts, or envisaging future scenarios (e.g., Doehring et al. 2023; Liguori et al. 2021; Vigliano Relva and Jung 2021). They therefore have a potential role to play in a range of aspects of adaptive EWM such as: (i) informing objectives and priorities, (ii) as a monitoring or research method to assess particular outcomes, (iii) communicating outcomes, (iv) awareness and education programmes, (v) building collective empathy and understanding around the environmental and social issues facing both human and ecological communities, (vi) conflict management, and (vii) envisaging novel and transformative solutions to environmental and social issues that impact EWM.

## Conclusions

While holistic management of river-floodplain systems is advocated the pragmatic reality, in the biophysical sciences at least, is that objectives, management actions, and the evaluation of outcomes may be structured in a hierarchical framework that, at its lower levels, focuses on broad groupings of biota or processes such as vegetation, fish, waterbirds or connectivity (e.g., Gawne et al. 2020). Achieving a more nuanced and sophisticated understanding of human values is a key element for generating sustained engagement and social license to implement changes in management (Moggridge and Thompson 2023). River-floodplain systems are highly valued by a range of people from a diversity of regions, livelihoods and cultures (Anderson et al. 2019; Moggridge and Thompson 2021). These ‘human-flow relationships’ (Anderson et al. 2019) are diverse and incorporate values such as human wellbeing (White et al. 2020), fishing and agriculture (Chowdhury and Moore 2017), recreational uses (Venohr et al. 2018), spiritual needs (Lokgariwar et al. 2014), cultural identity (Moggridge and Thompson 2021) and more (Anderson et al. 2019). Understanding such relationships is important to informing sustainable and just management of water (Anderson et al. 2019).

Our survey indicates NWV, and RWFs more broadly, are valued for a range of reasons. This includes the provision of ecological functions and services, such as regulating functions, the provision of habitat, biodiversity and intrinsic value, as well as the value of experiential interactions and the way these interactions with nature make people feel. This study highlighted that the way in which values are expressed is likely to vary depending on the framing of the question. The use of ecosystem functions and services in management planning, such as EWM, provides a useful common language for identifying objectives or describing outcomes, and advances in CES are continually improving the way relational values are incorporated in the ecosystem functions and services framework (e.g., Fish et al. 2016b; Pastor et al. 2022). The experiential, emotive connections many people have with RWFs are significant in underpinning attitudes, beliefs and behaviours and are unlikely to be adequately captured in approaches that rely heavily on assigning value through economic or willingness-to-pay mechanisms or focus strongly on instrumental values. As stated by Rodríguez-González et al. (2022) “*this challenge* [of better understanding and integrating the social dimensions of riparian vegetation] *includes a deeper reflection on how to study those social elements (e.g.,, which indicators, which methods)”*.

Accepting different narratives or values, identifying points for engagement, and collectively reaching mutually beneficial outcomes is largely about self-reflection and collective learning (Allan and Watts 2022; Liguori et al. 2021; Vigliano Relva and Jung 2021). We encourage readers to reflect on the values highlighted in this and other studies, and to consider the role of values in contested narratives and environmental management decisions in river-floodplain systems.

### Supplementary Information

Online-Resource-1-survey-questions

Online-Resource-2-supporting-information

## Data Availability

Non-identifiable data, such as raw text responses, is available in the associated online resources or from the corresponding author on request.

## Supplementary information

The online version contains supplementary material available at 10.1007/s00267-023-01900-2. Two pieces of supplementary material have been provided: Online Resource 1: survey questions and Online Resource 2: supporting information, which includes graphs of results to closed-format questions mentioned as text along with raw text responses (with no identifying information) to open-ended survey questions.
