# Supplementary material for: More Than a Service: Values of Rivers, Wetlands and Floodplains Are Informed by Both Function and Feeling
Source: Environ Manage. 2023 Oct 27;73(1):130–43. doi: 10.1007/s00267-023-01900-2 (PMC10786729; doi:10.1007/s00267-023-01900-2)
Supplement: Supplementary file 1 — Online-Resource-1-survey-questions [file 267_2023_1900_MOESM1_ESM.pdf]

Online Resource 1 – survey questions

Article title: More than a service: values of rivers, wetlands and floodplains are informed by both function and feeling

Journal: Environmental Management

Authors and affiliations: Cherie J. Campbell[1✉], Siwan Lovett[2], Samantha J. Capon[3], Ross M. Thompson[1], Fiona J. Dyer[1]

✉ Cherie Campbell, Centre for Applied Water Science, Institute for Applied Ecology, Faculty of Science and Technology, University of Canberra, Bruce, Australian Capital Territory, 2601. Cherie.Campbell@canberra.edu.au

1. Centre for Applied Water Science, Institute for Applied Ecology, Faculty of Science and Technology, University of Canberra, Bruce, Australian Capital Territory, Australia 2601.
2. Australian River Restoration Centre, Canberra, Australian Capital Territory, Australia, 2601.
3. Australian Rivers Institute, Griffith University, Nathan, Queensland, Australia, 4111.

Start of Block: Introduction

Non-woody wetland and floodplain vegetation: values and functions provided

Introduction and background

Welcome and thank you for participating in this survey. This survey aims to better understand the values associated with non-woody wetland and floodplain vegetation (NWV) - basically all the plants you see in riverine areas - rivers, wetlands and floodplains - except for trees and large shrubs. The survey is Australian centric, focused around the Murray-Darling Basin, however perspectives from outside the Basin and/or Australia are also sought. This information will be used to help inform future watering decisions related to non-woody vegetation (NWV) in the Murray-Darling Basin.

This survey forms part of a PhD project aiming to characterise good condition for NWV. Having a greater understanding of how society values the role of vegetation in rivers, wetlands and floodplains will help to target outcomes that are supported by multiple sectors of the community. To achieve this requires a wide range of perspectives, from a broad spectrum of the community.

This survey is relevant to anyone with a love of riverine, wetland and floodplain environments. It is anticipated that the survey will take approximated 5 – 10 minutes to complete.

Please feel free to circulate this survey.

The survey is open and available to complete until the 9th May 2021.

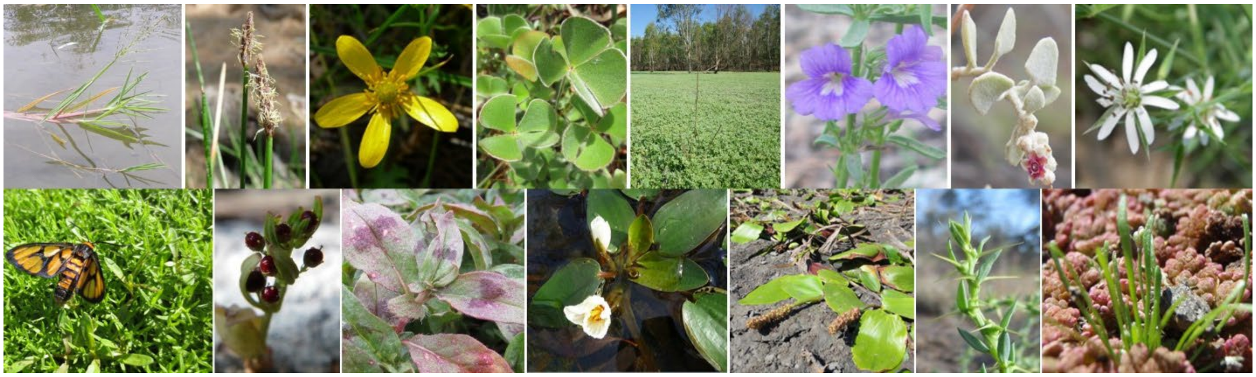

Page Break

Privacy, consent and ethics
This survey has been approved for release under University of Canberra ethics permit 4636. By reading this information and completing the survey you consent to be a participant. No identifying information is stored with the survey responses and responses will be collated and analysed anonymously.

Definitions
Non-woody wetland and floodplain vegetation (NWV) refers to all vegetation, except for large shrubs and trees, found in riverine areas - e.g. in and along rivers, and in wetland and floodplain habitats associated with rivers. It includes vegetation seen during all hydrological phases (e.g. when wet, partially wet and dry) and includes seedbanks (e.g. seed stored in the soil). The focus is on vascular vegetation (e.g. not algae) with the inclusion of Charophytes. Inland riverine, wetland and floodplain systems that are reliant on flooding are the target habitat types.

Survey outcomes
A summary of the survey outcomes will be made available on the Flow-MER website (https://flow-mer.org.au/) and distributed through the newsletter (click here to subscribe). If you would like to be contacted in relation to this survey, or receive a copy of the results, you can provide contact information in the final section. This information will be held separately to the survey responses.

Contact
For more information please contact Cherie Campbell, Centre for Applied Water Science, University of Canberra, cherie.campbell@canberra.edu.au, or visit the Flow-MER website (https://flow-mer.org.au/).

Consent

○ By clicking this box I agree to participate in this survey. I have read the information provided and consent to the data being used as described above (1)

End of Block: Introduction

Start of Block: Section 1: Context

Section 1: Context

This section of the survey collects information on your interest in and relationship with rivers, wetlands, floodplains and environmental water management

Q1.1 Please select ALL options that describe your interest in rivers, wetlands, floodplains, and environmental water management

- ☐ Amateur naturalist / environmentalist - e.g. birdwatching, botanist, appreciation of nature, etc
- ☐ Currently reside (or have previously lived) in a river-floodplain community
- ☐ Irrigator / farmer
- ☐ Professional - current or previous employment in environmental water management or research
- ☐ Professional - current or previous employment related to rivers, wetlands or floodplains (e.g. tourism, education, floodplain beekeeping, firewood collection, freshwater aquaculture, etc) (please specify)
- ☐ Recreational - e.g. fishing, camping, boating, canoeing, etc
- ☐ Student - study / research related to rivers, wetlands, floodplains or environmental water management
- ☐ Traditional custodian
- ☐ Other (please specify) ____
- ☐ Not interested in rivers, wetlands, floodplains, or environmental water management

Page Break

Q1.2 Please select ONE option to best describe your interest in rivers, wetlands, floodplains, and environmental water management

- ○ Amateur naturalist / environmentalist - e.g. birdwatching, botanist, appreciation of nature, etc
- ○ Currently reside (or have previously lived) in a river-floodplain community
- ○ Irrigator / farmer
- ○ Professional - current or previous employment in environmental water management or research
- ○ Professional - current or previous employment related to rivers, wetlands or floodplains (e.g. tourism, education, floodplain beekeeping, firewood collection, freshwater aquaculture, etc) (please specify)
- ○ Recreational - e.g. fishing, camping, boating, canoeing, etc
- ○ Student - study / research related to rivers, wetlands, floodplains or environmental water management
- ○ Traditional custodian
- ○ Other (please specify) ____
- ○ Not interested in rivers, wetlands, floodplains, or environmental water management

---

Q1.3 How would you describe your level of knowledge in relation to non-woody wetland and floodplain vegetation and environmental water management?

- ○ Expert (e.g. specific training and/or multiple years of professional or personal experience)
- ○ High level of knowledge
- ○ Moderate level of knowledge
- ○ Limited level of knowledge
- ○ No knowledge (e.g. no professional or personal experience)

End of Block: Section 1: Context

Start of Block: Section 2: Value and function of non-woody wetland and floodplain vegetation

Section 2: Value and function of non-woody wetland and floodplain vegetation

This section of the survey is interested in how you value non-woody wetland and floodplain vegetation and why.

Q2.1 Do you value non-woody floodplain and wetland vegetation?

- ◯ 5 - Very highly
- ◯ 4
- ◯ 3 - Moderately
- ◯ 2
- ◯ 1 - Not at all
- ◯ 0 - I don't know

Skip To: Q2.2 If Q2.1 Do you value non-woody floodplain and wetland vegetation? = 0 - I don't know

Skip To: Please expand on why you do not value non-woody wetland and floodplain vegetation? If Q2.1 Do you value non-woody floodplain and wetland vegetation? = 1 - Not at all

Display This Question:

If Q2.1 Do you value non-woody floodplain and wetland vegetation? = 3 - Moderately

Or Q2.1 Do you value non-woody floodplain and wetland vegetation? = 4

Or Q2.1 Do you value non-woody floodplain and wetland vegetation? = 5 - Very highly

For what broad reasons do you value non-woody wetland and floodplain vegetation?

____

____

____

____

____

Display This Question:

If Q2.1 Do you value non-woody floodplain and wetland vegetation? = 2

Or Q2.1 Do you value non-woody floodplain and wetland vegetation? = 1 - Not at all

Please expand on why you do not value non-woody wetland and floodplain vegetation?

____

____

____

____

____

Page Break

Q2.2 How important do you think non-woody wetland and floodplain vegetation is in supporting the following functions in rivers, wetlands and floodplains? Please see text below for definitions of the categories

	Extremely important	Very important	Moderately important	Slightly important	Not at all important	I don't know
Activities of commercial value	○	○	○	○	○	○
Activities of cultural value	○	○	○	○	○	○
Activities of educational value	○	○	○	○	○	○
Activities of recreational value	○	○	○	○	○	○
Aesthetic / visual appeal	○	○	○	○	○	○
Maintenance of biodiversity and ecological health	○	○	○	○	○	○
Provision of habitat	○	○	○	○	○	○
Regulation of environmental functions	○	○	○	○	○	○
Other (please specify)	○	○	○	○	○	○

Please elaborate or add any additional roles or functions you think non-woody wetland and floodplain vegetation provides?

____

____

____

____

____

Definitions:

Activities of commercial value - the contribution non-woody vegetation makes in supporting activities from which someone derives a financial benefit, such as tourism, beekeeping, photography, firewood collection, commercial yabby collection, etc.
Activities of cultural value- the contribution non-woody vegetion makes, for example, in supporting cultural and spiritual connections with country, in protecting cultural artifacts and places, in the maintenance and provision of culturally significant species for food, fibre, medicine and other purposes
Activities of educational value - the contribution non-woody vegetation makes to the provision of areas and / or plant / vegetation material for school excursions and scientific research
Activities of recreational value - the contribution non-woody vegetation makes in supporting activities such as camping, fishing, boating, canoeing, hunting, bushwalking
Aesthetic / visual appeal - the role non-woody vegetation plays in the way rivers, wetlands and floodplains look, the provision of attractive landscape features
Maintenance of biodiversity and ecological health - the role non-woody vegetation plays in supporting a wide variety of animals and plants and in maintaining the health of rivers, wetlands and floodplains
Provision of habitat - the role non-woody vegetation plays in providing areas to feed, breed, and live for a wide range of animals (e.g. birds, fish, turtles, small mammals, insects, reptiles, etc.)
Regulation of environmental functions - the role non-woody vegetation plays in regulating environmental functions such as water quality, erosion control, runoff and nutrient uptake

End of Block: Section 2: Value and function of non-woody wetland and floodplain vegetation

---

Start of Block: Section 3: Environmental flows and non-woody wetland and floodplain vegetation

Section 3: The role of environmental flows

This section of the survey is interested in how you value the use of environmental flows to manage non-woody wetland and floodplain vegetation in rivers, wetlands and floodplains. Environmental flows are managed changes in river water discharge (e.g. dam releases, pumped water, opening or closing regulators, weir pool raising or lowering) that is intended to maintain or improve the health of rivers, wetlands or floodplains.

Q3.1 Do you think environmental flows should be used to manage non-woody wetland and floodplain vegetation?

- ◯ 5 - To a great extent
- ◯ 4
- ◯ 3 - To some extent
- ◯ 2
- ◯ 1 - Not at all
- ◯ 0 - I don't know

Skip To: End of Block If Q3.1 Do you think environmental flows should be used to manage non-woody wetland and floodplain vegetation = 0 - I don't know

Skip To: Please expand on why you don't think environmental flows should be delivered to non-woody wetland and floodplain vegetation If Q3.1 Do you think environmental flows should be used to manage non-woody wetland and floodplain vegetation = 1 - Not at all

---

Page Break

Q3.2 Please indicate your support of the delivery of environmental flows to non-woody wetland and floodplain vegetation for the following reasons. Please select ALL relevant options. Please see text below for definitions of the categories. There is space below to provide details of the specific activities or functions you would like to see supported.

	Strongly support	Moderately support	Neither support or disagree	Moderately disagree	Strongly disagree	I don't know
For aesthetic / visual appeal	○	○	○	○	○	○
To maintain or improve biodiversity and ecological health	○	○	○	○	○	○
To provide habitat	○	○	○	○	○	○
To regulate environmental functions	○	○	○	○	○	○
To support activities of commercial value	○	○	○	○	○	○
To support activities of cultural value	○	○	○	○	○	○
To support activities of educational value	○	○	○	○	○	○
To support activities of recreational value	○	○	○	○	○	○
Other (please specify)	○	○	○	○	○	○

Please provide details of the specific activities or functions you would like to see supported

____

____

____

Definitions:

For aesthetic / visual appeal - to support the way rivers, wetlands and floodplains look, to provide attractive landscape features
To maintain or improve biodiversity and ecological health - to support a wide variety of animals and plants and to maintain the health of rivers, wetlands and floodplains
To provide habitat - to provide areas to feed, breed, and live for a wide range of animals (e.g. birds, fish, turtles, small mammals, insects, reptiles, etc.)
To regulate environmental functions - to support non-woody vegetation in regulating environmental functions such as water quality, erosion control, runoff and nutrient uptake
To support activities of commercial value - to support activities from which someone derives a financial benefit, such as tourism, beekeeping, photography, firewood collection, commercial yabby collection, etc.
To support activities of cultural value- to support, for example, cultural and spiritual connections with country, to protect cultural artifacts and places, to maintain or provide culturally significant species for food, fibre, medicine and other purposes
To support activities of educational value - to support the provision of areas and / or plant / vegetation material for school excursions and scientific research
To support activities of recreational value - to support activities such as camping, fishing, boating, canoeing, hunting, bushwalking

---

Display This Question:

If Q3.1 Do you think environmental flows should be used to manage non-woody wetland and floodplain v... = 1 - Not at all

Please expand on why you don't think environmental flows should be delivered to non-woody wetland and floodplain vegetation

____

____

____

____

____

End of Block: Section 3: Environmental flows and non-woody wetland and floodplain vegetation

---

Start of Block: Additional comments

Stories and thoughts

Please share any stories, thoughts, or memories about rivers, wetlands and floodplains that illustrate their value and importance to you. For example, characteristics of your favourite place along the river (e.g. grassy plains, flowers, or tall reeds), particular species you value, or favourite locations or activities.

____

____

____

____

____

If you would like to make any additional comments not captured elsewhere, please do so here.

____

____

____

____

____

End of Block: Additional comments

Start of Block: Survey outcomes and contact details

Please provide contact details if you would like to receive a copy of the survey outcomes or are happy to be contacted in relation to this survey.

- ○ Name ____
- ○ Email ____
- ○ Phone ____

End of Block: Survey outcomes and contact details

Start of Block: Thank you

Thank you for completing this survey, it is greatly appreciated. I value the time taken and the willingness to share your thoughts.

Please feel free to circulate the survey.

A summary of the survey outcomes will be made available on the Flow-MER website (https://flow-mer.org.au/) and distributed through the newsletter (click here to subscribe).

For additional information please contact Cherie Campbell, Centre for Applied Water Science, University of Canberra, cherie.campbell@canberra.edu.au.

For information about the Flow-MER project please visit https://flow-mer.org.au/

End of Block: Thank you
