# Supplementary material for: More Than a Service: Values of Rivers, Wetlands and Floodplains Are Informed by Both Function and Feeling
Source: Environ Manage. 2023 Oct 27;73(1):130–43. doi: 10.1007/s00267-023-01900-2 (PMC10786729; doi:10.1007/s00267-023-01900-2)
Supplement: Supplementary file 2 — Online-Resource-2-supporting-information [file 267_2023_1900_MOESM2_ESM.pdf]

Online Resource 2 – supporting information

Article title: More than a service: values of rivers, wetlands and floodplains are informed by both function and feeling

Journal: Environmental Management

Authors and affiliations: Cherie J. Campbell[1]✉, Siwan Lovett[2], Samantha J. Capon[3], Ross M. Thompson[1], Fiona J. Dyer[1]

✉ Cherie Campbell, Centre for Applied Water Science, Institute for Applied Ecology, Faculty of Science and Technology, University of Canberra, Bruce, Australian Capital Territory, 2601. Cherie.Campbell@canberra.edu.au

1. Centre for Applied Water Science, Institute for Applied Ecology, Faculty of Science and Technology, University of Canberra, Bruce, Australian Capital Territory, Australia 2601.
2. Australian River Restoration Centre, Canberra, Australian Capital Territory, Australia, 2601.
3. Australian Rivers Institute, Griffith University, Nathan, Queensland, Australia, 4111.

Purpose of the document

This document provides supporting information for survey questions addressed in the main body of the paper (see details above). This includes graphs of results to closed-format questions mentioned as text along with raw text responses (with no identifying information) to open-ended survey questions. A copy of the survey questions is also provided in Online Resource 1. The main body of the paper uses data from a sub-set of these questions to address the research question: ‘what is the value of non-woody vegetation (NWV) in rivers, wetlands and floodplains (RWFs)?’

Section 1: Context

Q1.1 Please select ALL options that describe your interest in rivers, wetlands, floodplains, and environmental water management* and *Q1.2 Please select ONE option to best describe your interest in rivers, wetlands, floodplains, and environmental water management

More than 70% of respondents identify with more than one type of interest or relationship with rivers, wetlands, floodplains (RWFs) and environmental water management (EWM), with two percent identifying with six of the 10 options offered (Figure S.1).

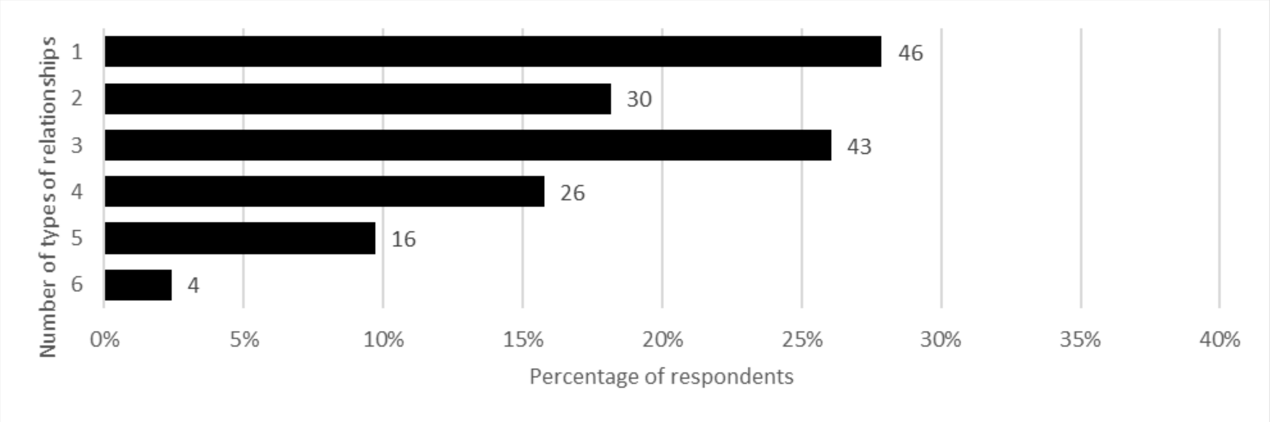

Figure S.1 Percentage of respondents who identified as having multiple interests or relationships with rivers, wetlands, floodplains, and environmental water management. Ten options for different interests were provided (including 'other') along with 'not interested in rivers, wetlands, floodplains or environmental water management', n = 443 selections from 165 respondents. The number at the end of each bar is the number of respondents who selected the corresponding number of relationships.

The three most common interests or relationships were amateur naturalist / environmentalist (23%), recreational (22%) and professional involvement related to EWM or research (20%) (Figure 1 in the main body of the paper). In terms of identifying with one interest or relationship, respondents predominantly identified with professional involvement related to EWM or research (39%) and amateur naturalist / environmentalist (30%) (Figure S.2).

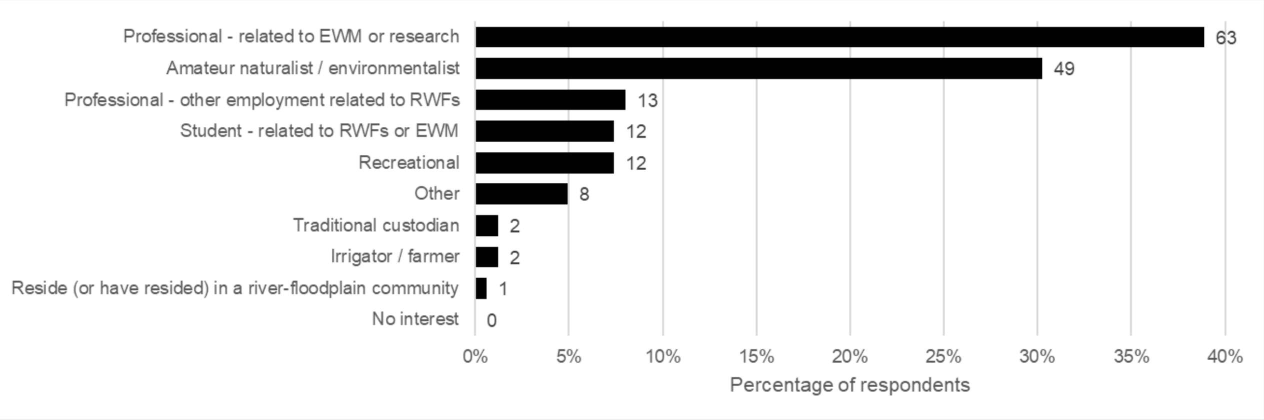

Figure S.2 Number and percentage of respondents who selected ONE option to best describe interests or relationships with rivers, wetlands, floodplains (RWFs) and environmental water management (EWM), n = 162 respondents.

Nineteen (Q1.1 allowing multiple options) and eight (Q1.2 restricted to one option) respondents provided additional details in terms of their interest or relationship with RWFs and EWM (Table S.1).

Table S.1 Text responses to the category 'Other' for questions 1.1 (multiple options) and 1.2 (one option).

#	Responses to 'Other' for questions Q1.1 (multiple options) and Q1.2 (one option)
	Q1.1 (multiple options)
1	Photography
2	Landcare volunteer - mostly regeneration on creeks and rivers locally
3	Artist of Australian plants and habitats with interest in different eco systems
4	wildlife conservation
5	I am a Yorta Yorta woman belonging to the Yorta Yorta people. I live on the Murray River and I witness Country daily. My interest in rivers is purely because my bloodline connects me to Country, to water and to my people. To feel and see the rivers (Murray, Goulburn, Campaspe. Ovens, Broken etc), wetlands (Barmah, Moira etc), floodplains makes me feel happy, well what is left of them from what the old people talk about.
6	Volunteer with wetland care group and Waterwatch citizen science program.
7	recreation: curiosity, aesthetics and photography
8	previous internship in limnological research group financed by an inland fishery
9	Essential environmental service that supports life.
10	Staff wetland biologist for an environmental consulting firm
11	Landowner of conservation covenant areas working with our neighbour to re-establish our riparian areas as healthy, complex and functional wetland habitats.
12	all of the above
13	algaculture
14	Manage wetland projects for threatened species
15	Volunteer work to do with rivers
16	I want to say LOVE and NEED (as in dependence for life) which I imagine feeling when I am present in a wetland and don't seem adequately represented by the categories of amateur naturalist/environmentalist or recreation.
17	landholder with creek frontage (not irrigator or farmer)
18	Intrinsic value of the environment
19	recreational fisher / duck hunter
	Q1.2 (one option)
1	I also want to select currently reside in river-floodplain community, environmentalist and recreational :-)
2	Yorta Yorta Country: The way in which I connect, see, feel, hear, taste, sound connect (sing, language) and speak of Country is essential to the way in which the water must be there for the rivers, wetlands, floodplain and biodiversity. If it is no longer there, then I will become ill and upset. Seeing Country from the sky brings sadness. There is not much left.
3	recreation: curiosity, aesthetics and photography
4	Essential to environment and hence my own survival
5	Manage project for wetland bird species
6	Volunteer
7	I want to say LOVE and NEED (as in dependence for life) which I imagine feeling when I am present in a wetland and don't seem adequately represented by the categories of amateur naturalist/environmentalist of recreation.
8	Intrinsic value of these environments

Q1.3 How would you describe your level of knowledge in relation to non-woody wetland and floodplain vegetation (NWV) and environmental water management?

Most respondents are at least moderately knowledgeable in relation to NWV and EWM (72%). The greatest proportion of respondents identified as having a moderate level of knowledge (39%), with 34% indicating a high or expert level of knowledge. Less than 3% indicated they had no knowledge and 25% identified as having a limited level of knowledge (Figure S.4).

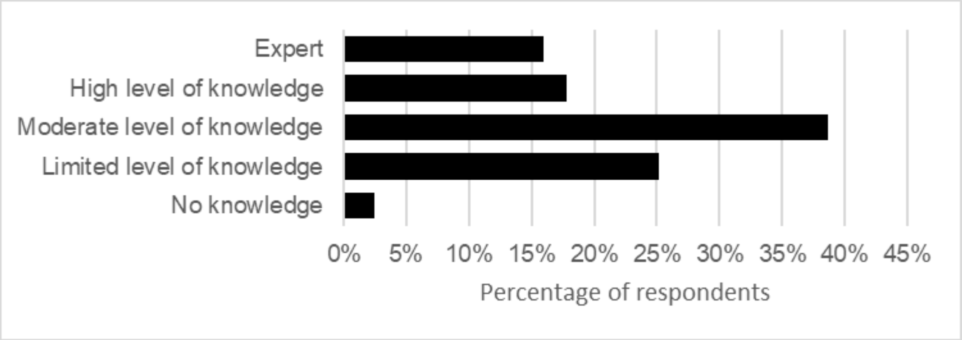

Figure S.3 Self-identified level of expertise of survey participants in relation to non-woody wetland and floodplain vegetation and water management, n = 163 respondents.

Section 2: Value and function of non-woody wetland and floodplain vegetation

Q2.1 Do you value non-woody floodplain and wetland vegetation?

The majority of respondents (71%) value NWV very highly (5/5), with virtually all respondents (99.4%) indicating a moderate or greater value for NWV (i.e. 3, 4 or 5 out of 5). A single respondent indicated 'I don't know' (Figure S.5).

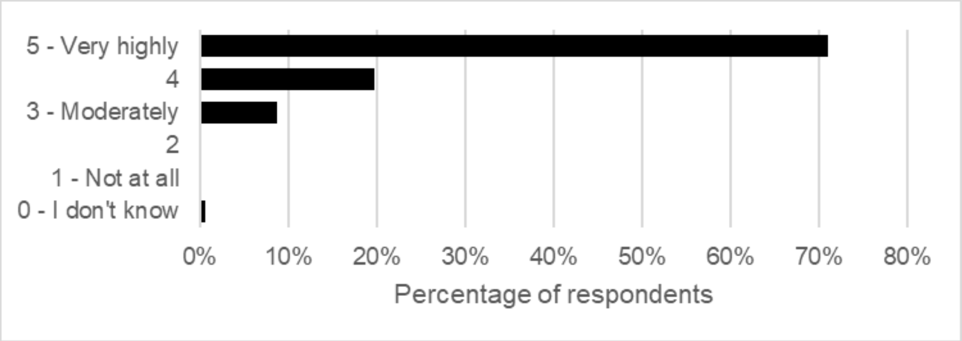

Figure S.4 Percentage of respondents who value non-woody wetland and floodplain vegetation on a scale from '5 – very highly' to '1 – not at all' (including '0 – I don't know'), n = 162 respondents.

For what broad reasons do you value non-woody wetland and floodplain vegetation?

One hundred and thirty-nine survey participants provided text responses to explain the reasons they value NWV. In line with recognised qualitative research approaches (e.g. Elo and Kyngaes 2008, de Casterle, De Vliegher et al. 2021) text responses were read and reread to identify common themes prior to the systematic coding of responses against identified nodes in NVivo 12 Pro. Initial assessment of common themes in these responses indicated good alignment with ecosystem functions and services categories used in questions Q2.2 and Q3.2 in this survey (see Online Resources 1). Following ideas in Ives and Kendal (2014) we were also interested in the distinction between assigned values (i.e. the values that people assign to things in the world or their relative worth, e.g. monetary value) and underlying values (i.e. values which shape people's perceptions of the world e.g. biocentric, social-altruistic). Therefore, following the initial process of reading and rereading, text responses, or parts of responses, were coded against two separate identified groups of themes: i) value orientations (Table S.2) and ii) ecosystem functions and values (Table S.3).

Value orientations

Values orientations refers to the underlying values held by people which shape their perceptions of the world (e.g. Ives and Kendal 2014). For our purposes we recognized the categories of biospheric, social-altuistic, hedonic and egoistic as defined in Table S.2 based on reference to Steg and de Groot (2012), and Bouman, Steg et al. (2018). It should be noted the survey question 2.1 ('For what broad reasons do you value non-woody wetland and floodplain vegetation?') was not specifically designed to elicit different value orientations and there are limitations in trying to interpret the underlying value orientation from the value statements given (see footnote under Table S.2).

Table S.2 Value orientations, definitions and guidelines used to code text responses to question 2.1.

**Value orientations**[+]	**Definitions** (as applied in this study)	**Guidelines** (for assigning value statements; see also limitations^)
Biospheric	Nature-focused; values align with environmental factors with no obvious link to humans (others or self)	Included statements that referred to environmental values or benefits to plants, animals or ecosystems with no explicit reference to humans (others or self)
Hedonic	Individual-focused; values align with individual feelings of positivity and pleasure	Included personal references to feelings of pleasure, wellbeing, enjoyment, etc. also included references to aesthetics and benefits to mental health.
Social-altuistic	Human-focused; values align with the welfare and well-being of other humans	Included references to potential/perceived benefits to humans, but without an explicit reference to the individual (e.g. references to agriculture, cultural values or aboriginal land management, and general references to recreational or social benefits or aiding management)
Egoistic	Individual-focused; values align with personal resources, power, achievement	Required explicit reference of concern or benefit to personal resources or individual commercial gain

+Value orientations based on information in Steg and de Groot (2012), Ives and Kendal (2014), and Bouman, Steg et al. (2018)
^Limitations: question 2.1 was an open-ended text response. It is therefore difficult to know the intent or value orientation behind many of the statements. For example, statements of ecosystem services, such as “stops wind erosion”, “water filtration”, or “protection from storm surges” may be biospheric (simply valuing the process for its contribution to functioning ecosystems), or the value orientation may be social-altuistic or egoistic (e.g. concerned about the impact of erosion, water quality or storms on others or their own resources). Similarly, broad value statements concerning potential benefits to agriculture, water and food security, and recreation may be social-altuistic (concerned about the impact on others) or egoistic (concerned about the impact to personal resources). The above guidelines were followed to provide consistency only within this study.

From the 139 responses to the question ‘For what broad reasons do you value non-woody wetland and floodplain vegetation?’ 203 individually coded values were identified against categories of value orientations. The percentage of values coded against the different value orientations is given in Figure S.6. The raw text responses are provided in Table S.5.

The majority of responses (65%) appear to align with a biospheric value orientation, followed by hedonic (24%) and social altruistic (12%). However, as mentioned in the footnote to Table S.2, there are limitations in the ability to know the intent or value orientation behind many of the statements. Because there are a high proportion of statements that relate to ecological functions, such as regulating functions, habitat provision and biodiversity (see Figure 2 in the main body of the paper), this, according to our definitions and guidelines in Table S.2, corresponds to a high proportion of coded values aligning with a biospheric value orientation. A number of these regulating functions (e.g. water filtration), that have been included here under biospheric value orientation, may indeed be valued for their benefit to nature (e.g. water quality for biota such as frogs - biospheric), or they may be valued for their benefit to (other) humans (e.g. improved water quality for children to enjoy swimming – social-altuistic) or to the individual, either in terms of their own resources (e.g. potable water for stock and domestic use – egoistic) or personal pleasure (e.g. pleasure derived from seeing or listening to frogs – hedonic). Despite these limitations, the key point is that respondents perceive the value of NWV in multiple ways: for its intrinsic worth or benefit to other organisms or ecological processes (biospheric), in promoting feelings of pleasure, positivity, well-being, or beauty within an individual (hedonic), and for the benefits (real, potential or perceived) it provides to humans (social-altuistic) and potentially to the individual (egoistic).

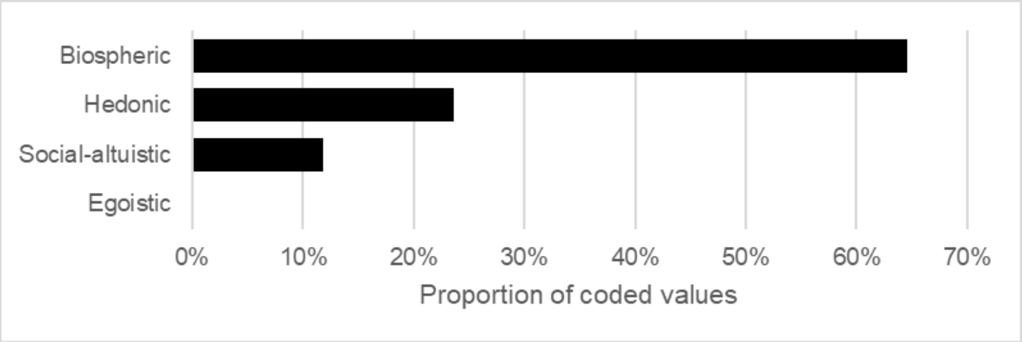

*Figure S.5 Proportion of values identified in response to the question ‘for what broad reasons do you values NWV’ that align with categories of value orientations. *Categories of value orientation were defined a priori (defined in Table S.2), n = 203 coded values from 139 responses.*

Ecosystem functions and values

The ecosystem functions and values categories used in this survey are based on de Groot, Wilson et al. (2002), and Capon, Chambers et al. (2013) with a number of notable alterations (see also Table S.3):

- The inclusion of 'maintenance of biodiversity and ecological heath'; this was included to recognize the intrinsic value of NWV, and RWFs more broadly, and is not captured by the ecosystem services approach
- The category of 'production' was not explicitly included and was adapted for the purposes of this survey to be 'activities of commercial value – defined as being the contribution NWV makes in supporting activities from which someone derives a financial benefit, such as tourism, beekeeping, photography etc.
- The information category of spiritual and historic information was not explicitly included and was assumed to be covered by the cultural information category; we acknowledge this isn't well-reflected in the definition.

The ecosystem functions and values categories were defined *a priori* except for 'Wellbeing-emotional connection'. This category was identified and added as an additional category following analysis of the data to open-ended text questions. Guidelines are provided for assigning text responses against the different categories (Table S.3) and sub-categories within the regulating function category (Table S.4).

From 139 responses to the question 'For what broad reasons do you value non-woody wetland and floodplain vegetation?' 373 individually coded values were identified against ecosystem functions and values categories, with an average of approximately three values identified per response, though this ranged from 1 to 10. The percentage of values coded against the different ecosystem functions and values themes is given in Figure 2 in the main body of the paper. For values within the regulation of environmental functions theme this is further defined according to groupings of regulating functions (Figure 3 in the main body of the text). The raw text responses are provided in Table S.5. The findings are interpreted and discussed in the main body of the paper.

Table S.3 Categories of functions and services, definitions, and guidelines used to code text responses to question 2.1 and the question 'please share any stories, thoughts, or memories about rivers, wetlands and floodplains that illustrate their value and importance to you'

**Functional categories⁺**	**Alignment to values categories in survey**	**Definitions** (as provided in the survey)	**Guidelines** (for assigning value statements)
Regulation	Regulation of environmental functions	The role NWV plays in regulating environmental functions such as water quality, erosion control, runoff and nutrient uptake	Included generic mentions of ecological or ecosystem or environmental function or service as well as references to specific regulating functions
Habitat	Provision of habitat	The role NWV plays in providing areas to feed, breed, and live for a wide range of animals (e.g. birds, fish, turtles, small mammals, insects, reptiles, etc)	Included references to habitat provision, either generically (e.g 'habitat') or in terms of the provision of feeding-foraging, or breeding habitat along with any mention of habitat corridors-connectivity.
Not applicable	Maintenance of biodiversity and ecological health	The role NWV plays in supporting a wide variety of animals and plants and in maintaining the health of rivers, wetlands and floodplains	Included references to biodiversity or diversity, intrinsic value, and any generic references to ecology or broad environmental health. For the 'stories' question this also includes responses that listed specific species or places.
Production^	Activities of commercial value	The contribution NWV makes in supporting activities from which someone derives a financial benefit, such as tourism, beekeeping, photography etc.	Included specific mention of values or activities with a commercial (financial) benefit (e.g. agricultural purposes, tourism)
Information	Aesthetic / visual appeal	The role NWV plays in the way rivers, wetlands and floodplain look; the provision of attractive landscape features	Included references to aesthetics, amenity, visual appeal, beauty, sounds, etc.
Information	Activities of recreational value	The contribution NWV makes in supporting activities such as camping, fishing, boating, canoeing, hunting, bushwalking	Included references to recreational pursuits such as hiking, canoeing, walking, fishing, birdwatching, etc along with generic references to recreation
Information	Educational purposes	The contribution NWV makes to the provision of areas and/or plant/vegetation material for school excursions and scientific research	Included references to experiences or opportunities linked to work, research, school, or other opportunities to learn, along with values associated with management (e.g. as monitoring indicators)
Information	Cultural purposes	The contribution NWV makes, for example, in supporting cultural and spiritual connections with country, in protecting cultural artifacts and places, in the maintenance and provision of culturally significant species for food, fibre, medicine and other purposes	Included references to aboriginal or indigenous uses or values
*Information	*Wellbeing-emotional connection	No definition was provided in the survey. This category was added a posteriori	Included responses that focus on the way NWV and RWFs more broadly made people feel (e.g. surprise, amazement, joy, peace, calm, etc.). It also includes explicit reference to mental health and wellbeing, along with nature interactions and nature play

Footnote to Table S.3: + Functional categories based on de Groot, Wilson et al. (2002), and Capon, Chambers et al. (2013); ^For the purposes of this survey we created the category 'activities of commercial value' and acknowledge that financial benefit is not mentioned as part of the original description of the 'production' category in de Groot, Wilson et al. (2002). *All categories were defined a priori except for Wellbeing-emotional connection which was added following analysis of text responses.

Table S.4 Categories of regulation functions, definitions, and guidelines used to code text responses to question 2.1

**Regulation functions**	**Description**	**Guidelines** (for assigning value statements)	**Note**
Gas regulation – carbon sequestration	Role of NWV in bio-geochemical cycles including carbon sequestration	Includes references to respiration, carbon sequestration, or CO_2 fixation	Included in de Groot, Wilson et al. (2002)
Climate regulation	Influence of NWV cover on climate (e.g. local temperature)	Includes references to effects on temperature, shading, local climate etc.	Included in de Groot, Wilson et al. (2002)
Disturbance prevention	Dampening of environmental disturbances e.g. floods, storms) by riparian, wetland and floodplain vegetation	Includes references to flood buffer zones, natural flood or flow management, protection from storm surges	Included in de Groot, Wilson et al. (2002)
Water regulation-supply	Influence of NWV in regulation and filtering of runoff and river discharge	Includes references to water quality, purity, filtering, filtration, treatment, clean water, water security,	Water regulation and water supply are combined here; they are recognized as separate categories in de Groot, Wilson et al. (2002)
Soil retention-formation	Role of vegetation root matrix and soil biota on soil retention; role in erosion and deposition, organic matter accumulation, decomposition	Includes references to erosion, soil protection, bank, soil, substrate stability, soil maintenance, healthy soil	Soil retention and soil formation are combined here; they are recognized as separate categories in de Groot, Wilson et al. (2002)
Nutrients-primary production[+](PP) -food webs	Role of NWV in nutrient storage and recycling, and in primary production and food webs in energy exchange between aquatic and terrestrial systems	Includes reference to food web, food chain, primary producer, production or productivity, nutrient cycling, exchange of energy etc.	de Groot, Wilson et al. (2002) recognizes nutrient regulation and biological control (through trophic-dynamic relations) and Capon, Chambers et al. (2013) additionally recognize energy transfer
Ecological stability-resilience	Role of NWV in maintaining the resilience or stability of ecosystems	Includes reference to ecological stability or resilience	Not recognized in de Groot, Wilson et al. (2002)
Generic (unspecified)	Not applicable	Includes generic mentions of ecological or ecosystem or environmental function or service where allocation to a specific regulating function was not possible	Not applicable to de Groot, Wilson et al. (2002)

Footnote to Table S.4: [+]We acknowledge primary production is likely covered in de Groot, Wilson et al. (2002) under the high-level category production functions

Table S.5 Text responses to the descriptive part of question 2.1 'For what broad reasons do you value non-woody wetland and floodplain vegetation?'

#	For what broad reasons do you value non-woody wetland and floodplain vegetation?
1	An indicator of condition (for biodiversity). This vegetation is essential for maintaining biodiversity and associated habitats, with different species occupying different draw down zones or in permanent water.
2	Halts erosion, fish and other fauna habitat, bird feeding ground
3	Ecologically for the diversity, habitat, services and functions NWV provides, personally for the aesthetics, peace and creative space well-vegetated wetland/floodplains offer
4	Core component of ecosystem function and primary producer at the base of the foodchain.
5	They are home to hundreds of native plants and animals
6	The bird and plant life it supports!! Appreciating these aspects of nature makes my heart sing!!
7	A love of biodiversity and the colour green! I've spent most of my life in hot dry places where the boom in wetland plant and animal life after big rainfall events was an amazing contrast to the usual stark landscape.
8	Farmer's daughter and amateur conservationist appreciating the value of the wetland for agricultural purposes and also for the local and migratory birds and wildlife.
9	Habitat values/biodiversity; rivers/waterways provide excellent basis for corridor connectivity through/across the landscape; water quality - clean water for all living things;
10	For the protection of soil and animal, plant and bird life that inhabit the areas<br>And I think it helps the purity of the waterways.
11	It is part of the ecology of a place or location.<br>It is under pressure from the impacts of development and I want to see these areas protected for the future.<br>It is valuable as a natural resource.
12	Biodiversity, ecological stability and personal appreciation
13	I value all nature and enjoy hiking in various types of terrain. I therefore want to help conserve where I can.
14	Creates waterbird habitat
15	Environmental, habitat for birds/animals/fish, a relaxing environment to view,
16	Continuation of a healthy eco system and flourishing flora and fauna
17	Natural cycles, flora and fauna
18	To maintain native species biodiversity
19	water quality, aquatic life, environmental
20	Habitat value, beauty, function in filtering water, preventing erosion, home to many waterbugs, fish, birds, frogs etc providing food for platypus...
21	The eco system benefits they bring to floodplains, bird and aquatic life, renewal of river systems and agricultural lands if well managed
22	ecosystem services
23	This vegetation important in reducing turbidity during floods, absorbing nutrients, nature conservation of vegetation communities and providing habitat for a wide range of vertebrates and invertebrate fauna
24	Need to retain these areas for river and environment health.
25	Aquafer quality Wildlife variety broader environmental impact on surrounding ecosystem Human impact through water consumption
26	biodiversity, water filtration
27	Role in the food chain<br>Cultural uses and values<br>Role in water quality<br>Role as habitat for native species
28	Stops wind erosion
29	Their functional role in ecosystems and contributions to biodiversity
30	It's pretty and they plays an important role in our ecosystems,

31	Habitat<br>Ecology
32	It provides habitat and food for different animals and birds
33	Critical component of the floodplain and riverine ecosystem
34	providing a lovely green environment when I am canoeing and fishing, both on water and land
35	NWV is an integral part of all wetland ecosystems providing habitat and resources for invertebrate and vertebrate species.
36	Provide important structural and functional attributes for a healthy floodplain and riverine ecosystem. Including food sources (bees, insects), refuge and microclimate shelter (birds, bats, mammals); breeding habitat (hollows, temperature/shelter); respiration and carbon moderation; cooling effect on region; migratory and dispersal pathways; breakdown of organic matter back into food web; productivity contributions when respond to rainfall; influx of carbon to river system
37	It's my home.
38	Wildlife habitat, bank and soil stabilisation, water filtration properties.
39	provides the understorey for the system. Aquatic vegetation is the primary production of lakes and wetlands and underpins the ecosystem function
40	Richness and diversity<br>Capacity to surprise (by presence; by recovery: by beauty)<br>Resilience
41	high biodiversity - genetic diversity storage, environment for endemites, natural carbon sequestration, water retention, flood buffer zone, nesting grounds for birds, important stops on migration routes, potentially exotic biochemistry of autotrophic microorganisms
42	Habitat provision, supporting ecosystem functioning, visual appeal
43	Aesthetics - there are some great plants that occur in wetlands and on floodplains.<br>Function - they provide important resources in the landscape.
44	Pollution mitigation, habitat, water store (especially during floods)
45	Important habitat for wetland specific species. Beautiful areas for walking and being in nature.
46	Provides the habitat for life for many forms of life. One source of CO2 fixation. Aesthetic appeal,
47	The native fauna it feeds or houses
48	water quality, habitat values
49	Flooding health of rivers
50	A place to wander or sit to ponder on my day, reconnect with nature and the land around me. It helps me put things in perspective. Looking at how the plants change each day, month, season. The way the insects go about their business between the plants and some dipping onto the water top. Gazing at the reflections on the water and watching the plants wave in the breeze. It centres me. Helps me to manage my low mental health and feel gratitude. Like no matter what is going on in my life, this place will be here, doing its thing, each and every day.
51	Importance of filtering nutrient, sediment, and contaminant inputs within floodplain systems; habitat for diverse fauna; protection from erosion/storm surges
52	existence values, ecosystem function, recreational activities, water purification
53	To help maintain the condition of the wetland such that wetlands can continue to deliver valuable ecosystem services, and to support biodiversity.
54	They can be used a baseline or used for future management, and to use techniques to restore
55	habitat, nutrient cycling, boom-bust, role in food webs
56	Improved habitat for better ecosystem functions<br>Water security<br>Recreation and visual enhancement of surrounding area<br>Food security
57	For preservation
58	views
59	Water quality improvers, shoreline stabilisation, habitat for animals

60	For the diversity, visual appeal, and functional roles they play in the ecosystem.
61	I understand that they can help clean the water. They provide amenity value to view and protection for fish.<br>Although sometimes get in the way of recreation
62	For its diversity<br>For its aesthetics, visual appeal<br>As habitat for a wide range of native vertebrates and invertebrates<br>For its role in ecosystem function
63	It plays important roles in water supply and quality, carbon sequestration, carbon and nitrogen cycles, minimising erosion, habitat and breeding for native species (including birds, fish, insects etc), tourism and recreation, Indigenous use, ecosystem resilience, etc
64	biodiversity, flood mitigation, water treatment, carbon bio-sequestration
65	Contribution to biodiversity and habitat for resident aquatic and non-aquatic fauna, for recreational enjoyment and intrinsic value
66	The beauty.<br>The function of keeping banks stable and water clean.
67	It is all part of a big picture to create a diversified and healthy environment for vegetation and wildlife
68	Importance as part of wetland ecosystems, provide habitat, food, refuge, water quality improvements and nutrient cycling etc
69	Biodiversity, habitat conservation
70	The environmental value and the natural beauty
71	They are part of the ecosystem. As this they provide carbon and nutrients to aquatic ecosystems especially when flooded. They can provide filtering of overland flows. They provide physical protection of soils and sediments. Also provide habitat for terrestrial species.
72	Contributions to ecosystem services for species diversity and water quality
73	Habitat for macroinvertebrates & fish. Amenity.
74	It can a benefit and pest
75	Important for water quality of rivers, aesthetics.
76	I know the essential roles these habitats play within the broad ecosystem.<br>Additionally, I'm also interested in amateur bird watching so I value these wetlands for this purpose as well.<br>Also, they're just beautiful to look at!
77	An essential component of a functioning riverine-floodplain ecosystem.
78	Part of our environment ecosystem. Integral part of the river
79	Provides important habitat for water dependant species including breeding and foraging habitat for waterbirds such as Aust bitterns and ibis. Supports different plants and animals than what is found in woody floodplain wetlands.
80	The aesthetic qualities complement the large objects in the landscape such as trees. I am also always amazed at the transition between wet and dry phases in inland wetlands.<br>Also the habitat value for other creatures such as frogs is very important for the ongoing presence of this group of organisms in the landscape.
81	For its contribution to the overall health of eco systems
82	Bank stabilisation, Habitat,
83	I value all natural habitats in Australia, for the opportunity to engage with nature and enjoy the diversity of our native flora and fauna. I am a keen hiker.
84	Support for seasonally diverse or ephemeral ecosystems, natural flood management (no engineering required!).<br>Exchange or redistribution of energy and nutrients. Aesthetics.
85	ecosystem services
86	beautiful & important for ecosystems & food chains
87	It includes a lot of plant communities that have value in their own right but are also critical habitat for a very large number of animals - invertebrate and vertebrate. The vegetation also has physical importance in moderating flows and water quality
88	Intrinsic value<br>Ecosystem services

	Biodiversity<br>Aesthetic
89	Firstly, for its intrinsic value, these plants are supremely adapted to their variable habitats and are fascinating, beautiful organisms. Secondly, for their many and often critical functions that they provide to these ecosystems.
90	Ecosystem services
91	All wetland vegetation contributes to healthy wetland ecosystems which support all terrestrial life.
92	being part of the eco system, non-woody wetland help keep the cycle moving forward
93	It acts as a filter for water quality purposes. It protects against erosion. It provides habitat to aquatic and terrestrial wildlife.
94	They are an integral part of floodplain ecosystems.<br>Some of them are also quite beautiful, and their resilience can be surprising.
95	Biodiversity, aesthetics, habitat, ecosystem functions
96	Supporting a complex ecosystem in the immediate environment and downstream and critical to maintaining biodiversity in a much broader area.
97	For the health of the areas and the environment generally.<br>For diversity<br>For the beauty to adds to our lives..
98	Wetland and flood plains are a key part of the global biosphere, and are under pressure globally by human development and agriculture. The more we know about them, the more we can intelligently control future use (or non use) of these areas
99	As a habitat for birdlife (& other natural life). As a part of the overall well-being of a river system. I also value small wetland areas (possibly not connected to a larger river system) which are habitat for diverse forms of life and can even be part of urban areas for (therapeutic) recreational walking and general interaction.
100	for the ecological processes
101	Breeding grounds for birds, fish macroinvertebrates
102	It is all part of the ecosystem, and plays an essential role in maintaining soils/sediments as well as providing food for animals.
103	Ecological role, particularly for aquatic ecosystems - soil and sediment stability, allochthonous inputs to wetlands and waterways, shade, food for fauna, habitat for aquatic, semi aquatic and terrestrial fauna... and aesthetics
104	The functional roles it plays in these ecosystems, such as structurally complex habitat it provides for aquatic and terrestrial fauna, nutrient cycling, substrate stabilisation, etc.
105	For its physical habitat forming and food web roles, soils and bank stabilisation roles, and aesthetic values
106	Aesthetic appeal, beauty. Important functional roles in the ecology of the system, including influence on nutrient and sediment fluxes, primary production, and secondary production, role as food, role as habitat
107	It's an important part of the ecosystem, increases sedimentation/decreases erosion, regulates temperature etc.
108	Form an important ecological community providing habitat and food resources among other ecological functions
109	biodiversity, recreation (camping), aesthetics
110	They are important for the ecosystem (water filtering, habitat for animals).
111	Biodiversity and ecosystem function.
112	I value this vegetation type for it's role in stabilising wetland and river channel structure, for biofiltration, and for providing habitat for aquatic/semi-aquatic fauna.
113	It is part of the ecosystem that forms wetland and floodplains. It contributes to the health and beauty of the whole.
114	Essential ecosystem services - not limited to biodiversity, provisioning, physical stabilisation of banks and floodplains, part of ecosystem function<br>Can be very beautiful - I love our native wetland plants!
115	Foodweb, slow erosion, beauty
116	Habitat and food sources for ecological community
117	Wetland and floodplain vegetation is essential habitat for biodiversity, particularly for many birds, frogs insects and fish species. Because of this, they provide many valuable community resources.<br>They also provide ways for peoples to interact with the ecosystem by providing many recreational activities such as bird watching, something that I quite enjoy.

118	They serve as a<br>* cleaning and filtration system for our water<br>* habitat for wildlife<br>* measuring tool for the health of the water system<br>* recreational use
119	Managing nutrient loads in freshwater systemsProviding valuable habitat for a range of organisms in freshwater systemsAesthetically pleasing
120	Fundamental for ecological function, provide structural diversity, habitat
121	Habitat<br>Photosynthesis<br>Food<br>Substrate for other processes
122	All areas of river system are very important. If you take a link out of a chain the river system just gets weaker. If only we looked after the land like the Aboriginal people our future would be good. Because of the new people to Australia, things are not so good global warming etc. But on a positive note - at least the war on the environment is going well
123	ecologically important for plants and animals, recreational use for human wellbeing, habitat for migratory birds - international responsibility
124	Biodiversity conservation. Enjoyment of richness of natural environment.
125	Provide habitat and food for wide range of aquatic and terrestrial animals, important role in supporting the food web, important role in maintaining physical condition of banks, aesthetically beautiful
126	Habitat for freshwater organism, slowing flows, reducing erosion, visual amenity
127	As a Wiradjuri supermarket / classroom
128	Biodiversity of these systems<br>Unique and rare species<br>Unique functions and life histories of plants from a wetting/drying systems.
129	Indicator of healthy wetland ecosystem, provide food and shelter for aquatic and water-dependent fauna, aesthetically pleasing.
130	cultural significants<br>productivity<br>biodiversity<br>food
131	This vegetation provides habitat for the juvenile stages of fish and crayfish, surfaces for biofilms and cover for the invertebrates that form the basis of the riverine food web (for both terrestrial and aquatic species).
132	aquatic vegetation and aquatic and terrestrial wetland vegetation are critical habitat and foodweb components on which waterfowl and fish depend. Waterfowl and fish are the components I value the most.
133	how it interacts with the rest of the eco system and the value it provides fish and bird species
134	The habitat it provides for wetland species
135	For its ecological functions and for the principle of strengthening biodiversity
136	These ecosystems are part of the earth in which we reside and are part of
137	I value them because they places that exist
138	That vegetation is a critical part of a high value biodiversity hotspot, critical to life cycles of multiple species with knock on effects to the entire ecosystem, including healthy soil.
139	They are an essential part of the floodplain ecosystem providing all manner of services from food, shelter, soil stability and productivity to aesthetic, cultural and social benefits.

Please expand on why you do not value non-woody wetland and floodplain vegetation?

There are no responses to this sub-question as no respondents selected a value of NWV of 2 or 1 in response to the quantitative part of Q2.1 (see Figure S.5).

Stories and thoughts

Please share any stories, thoughts, or memories about rivers, wetlands and floodplains that illustrate their value and importance to you. For example, characteristics of your favourite place along the river (e.g. grassy plains, flowers, or tall reeds), particular species you value, or favourite locations or activities.

Eighty respondents chose to share stories, thoughts or memories about rivers, wetlands and floodplains that illustrate their value and importance to them. The results are presented (e.g. Figure 4 in the main body of the paper) and discussed in the main body of the paper. Table S.6 provides the raw text responses.

Table S.6 Stories and thoughts shared by respondents to illustrate the value and importance of rivers, wetlands and floodplains to them

Where possible raw text responses have been provided in their entirety. Occasionally specific information has been excluded (e.g. [….]) if the authors' felt there was a risk of identification of individuals.

#	Stories and thoughts
1	The flush of life following significant rain or flow events; the landscape transforms and so does biodiversity.
2	birdwatching along local rivers while kayaking. See the contrast of natural riverside/banks beauty and diversity versus where stock are able to graze to waters edge - erode banks etc.
3	One of my first memories as a child was spending all day in a creek scooping up jars of tadpoles and holding them up to the light to look at them. Then we'd put them back and scoop up some more to see them at their different stages of development. It doesn't feel so easy to have these experiences anymore, which is a shame.
4	Kayaking through a reedy floodplain in QLD and seeing a large group of baby brown snakes swimming across in front of us.
5	Seeing platypus in the Merri river and Dad actually catching one when fishing for rainbow trout!<br>My kids riding the rapids of Campbells creek after rain.<br>Seeing the bird-life increase due to jigsaw farm rejuvenating wetlands<br>The wrens dancing and the frogs singing from tall reeds at Merri river and Campbell's creek<br>The amazing number of black swans in the flood plains through Fox How<br>Masses of frogs following rain backroads Ararat to Camperdown
6	I loved spending time exploring wetlands as a child, just discovering all the plants and creatures that lived in them. I used to make little reed baskets and collect tadpoles and invertebrate larvae to watch and rear. Favourite species are dragonflies, damselflies and frogs, plus Eleocharis and Nardoo.
7	I can remember growing up as a small child on a dairy farm and we had wetlands on our property and I recall all the birdlife and how it also kept the farm from drying out over the summer.
8	Working mainly on the Wingecarribee River, which feeds into the Warragamba Dam, I am very aware of the effects of controlling the amount of water in a river. Water is sent via the Shoalhaven Scheme from the Shoalhaven River in Kangaroo Valley and lifted up to the Fitzroy Falls Reservoir and Wingecarribee Dam. From there it can be sent east to serve Wollongong's drinking water needs or run down the Wingecarribee River for Sydney. The River water is often high and fast when it would naturally be slow and sluggish. It is also very much colder than naturally.
9	Living between the Hopkins and the Merri as a kid. Beautiful spaces. Fishing and boating on the river. Walking along the riverbanks. Enjoying the bird life.<br>The waterfalls on the Hopkins with the eels.
10	It is the idea of a natural place that is most appealing but intervention and management has become the norm. Is it still natural? I think the answer is yes, but subject to protocols for management that recognise and place a high value on the conservation of the natural qualities and character of the wetland.
11	I live watching the native flowers bloom and the seasons change.
12	All river floodplains are important for Bird Habitat
13	Growing up I use to fish with my dad in rivers, have family picnics etc.<br>relaxing places for to visit and take my

	Children to for walks, relaxation, meditation, look for life meaning, ponder in natural wonders, one of my tools in my tool kit for when I'm stressed,
14	Probably for me my favourite part of wetlands are the sounds, squarks, chirps, gentle rustling in the reeds. Familiar and unfamiliar sounds. An orchestra of sound, musicians largely unseen.
15	Lakebed Herbland within Ramsar lakes at the Hattah Lakes.
16	I grew up beside the Brisbane River which had periodic floods and understood at an early age that building indiscriminately on floodplains was not a good idea. Also saw the human and environmental cost of building on Brisbane's wetlands and estuaries.<br><br>Particularly appreciate the wetlands of Moreton Bay, the mangrove systems, the RAMSAR and other bird sites, the lowland rainforest etc
17	I have memories of wading through a wetland with clear waist deep water that had an amazing diversity of water plants and scattered lignum bushes with hundreds of ibis nests filled with cheeping half-grown chicks. It was an intense immersion in nature
18	Any river habitat that supports native wildlife and fish ecosystems is great for that reason alone, but also shows the health of the wider local environment.
19	There is a quiet little wetland along the Murray, I have been told was an Aboriginal market garden. The water is long and quiet and clear. And in it is the diversity of times past. There are leaves of feather and ribbon, flowers above and below. Quiet drifters too, duck above and fish below. Gentle but ever searching for the next morsel. A taste of how all these places should be.
20	Milfoil for swans to make nests
21	I really like being able to walk through the plants surrounding bodies of water, as it increases the feeling of being immersed in nature. It makes it easier when there is a dedicated path as you are not worried about damaging plants. A bit of information on the species surrounding the waters or why the habitat is the way it is also greatly improves the experience.
22	Riverland wetlands SA
23	Yanga National Park in flood was an absolute delight. Lots of reeds with swan nests nestled in them, water covered with nardoo, birds galore.<br><br>During flood periods I have fond memories of catching many yabbies in the Barmera, Cobdogla, Katarapko region. During cool periods going past Mildura to catch Murray Crayfish.<br><br>My parents took us on many holidays along the River Murray.<br><br>After a long hot day picking grapes or cutting apricots we spent many afternoons at Katarapko or Cobdogla swimming and fishing to cool down.<br><br>One of my fondest memories as a child was playing hide and seek and running around large clumps of weedy wood that I now know as lignum :-)
24	I have lived beside and in a major inland ephemeral wetland all my life witnessing the constant state/s of change in the perpetual boom and bust cycle of life. These ecosystems are incredible in their complexity and function, somehow defying the constant pressure of a highly variable climate and increasing human impacts. Similarly, on an individual basis so many species have complexities and associations that you cannot comprehend in a lifetime.
25	Environmental water has supported a number of small wetlands that are either far out on the floodplain or disconnected to the river channel by several kilometres. When you visit those refugia during a dry cycle - and you drive through dusty, bare brown paddocks and big open sky country - and you see the shimmer of water in the distance - you get a glimpse into what an oasis in the desert might look like....and when you arrive it's like stumbling into a parallel universe where thig big noisy party is in full swing and you weren't invited! Thousands of waterbirds seem to be able to share the limited resources for a brief period of time as things ebb and flow....
26	Every aspect of where my feet have travelled within our rivers, wetlands and floodplains is important to me, to characterise - places one area or species over another and I cannot characterise. That's my value.
27	I have lovely memories of seeing platypus in the river and bee eaters nesting in the exposed banks at a site where I did water quality monitoring. On one occasion after heavy rain when the river had burst its banks I saw a platypus taking advantage of the flood to forage among the grasses and riparian vegetation that were usually out of reach, Unfortunately this site is now being developed and much for the vegetation has been removed from the catchment area adjacent to it. I don't image these species will persist.

28	Stories to share? nothing comes to mind, - sorry<br><br>Characteristics of favourite place(s) ? it is easier to say what it isn't.<br>I don't have a list of 'favourite' places, or a list of geographic sites; the big-ticket special places (whether Kakadu or Macquarie Marshes or Coorong) are as wonderful to me as the little ones; and I am not purist either: modified systems can be as magical as non-modified ones.<br><br>What makes just about any wetland or riverbank or billabong special is: not being trashed by livestock or feral animals (horses, deer, rabbit, pigs); one that has a dynamic water level, preferably near natural but no essential; one that is not eroded or polluted; and is not receiving human rubbish<br><br>Favourite activities: exploring; poking around; working out what's happening; what has happened
29	woody swamps are easily my favourite environment, as the microbial diversity is high, which suits my research needs. they are also very calm and I love to relax there (if its windy enough to avoid mosquitoes). I also appreciate reeds, throughout my childhood, I used to fish for crayfish in reed basins
30	The Great Cumbung Swamp [...] I remember sitting by the Lachlan river nearby at night, under beautiful clear skies, and hearing all the wildlife activity in the reeds and river. I saw the effects of flow on plant zonation and the landscape, and met local farmers and community members. I hear the Swamp is not doing so well these days. I think the condition of these places reflects our national perspective on them. Wetlands and floodplains were once an iconic emblem of our country, but as a nation we no longer have any sense of the value of our environment except as a resource to be exploited. Wetlands and the habitat they provide are more important than ever, and we should do whatever we can to ensure their continued integrity.
31	Paddling the anabranches of the River Murray near the border between Vic, NSW and SA. Redgum woodland on the bank and a diversity of plants on the banks. A plethora of birds nesting, roosting, feeding. A productive, peaceful place - looks amazing, cool compared with the surrounding cropland and has a significant number of plants that change with the seasons.
32	Creeks and rivers are calming and it is along creeks and rivers (especially where there are waterholes to safely swim) that I most enjoy spending my recreational time. If money were not a factor I would live along a swimmable creek.
33	My interest in science began with a fieldtrip in high school to mangrove wetlands! It was a fantastic introduction to ecology and even though I'm now a freshwater scientist, mangrove wetlands still are much loved.
34	My family has often chosen river-side situations for camping, and the aesthetics provided by non-woody vegetation has usually been a significant factor in that choice,
35	jerrabomberra wetlands<br><br>Tumut Plains floodplain
36	Where I live, instead of concrete storm water drains we have wetlands. I have found walking along these rejuvenating, especially during times of low mental health which I experience intermittently. Seeing the changes in water level after heavy rain. Listening to the chirping and buzzing of life within the dense plant habitat. Watching the grasses and reeds sway.<br>Another strong memory I have is walking along a boardwalk at a wetland with my father. The two of us stopping and waiting quietly for a long time, then excitedly pointing out a platypus that emerged. It was so special.<br>When I was really little we would camp near a river in the middle of summer. There was a spot where the water slowed and pooled. One time, my Mum and I sat in the water and dragonflies rested on our shoulders and hands. They stayed so long my long hair started to dry!
37	I always think back to my time […] in southern Louisiana, USA. Louisiana experiences extreme rates of coastal landloss annually, as wetlands (largely saltmarsh) are converted to open water. This loss is due to a number of human activities (hydrological modifications to river systems, draining of wetlands, oil and gas exploration, etc.), human-induced and natural subsidence rates, and sea-level rise. The drastic negative impacts of this loss on various cultures, society, the economy, and the ecology of these coastal systems demonstrates the value of wetlands and reaffirms the importance of protecting and conserving them.
38	It is lovely to spot birds and fish in mangroves/wetlands as I go for a walk.<br>I love seeing the plants and flowers too.<br>The whole ecosystem is very rich and unique. Wetlands help me relax.
39	being on a floodplain during and soon after flow events is amazing, the diversity and abundance of herbaceous species that emerge after these events is something every Australian should witness
40	Seeing our wetland/riparian areas ebb and flow with the seasons and during the last drought has really highlighted the diversity of plants that come and go, plus the importance of those species for that ecosystem to remain resilient. Our revegetation projects have highlighted to me the complexity of these systems and how important it is to protect

	the variety of wetlands we have in Australia. It has allowed me to learn much more about the complexity of those ecosystems. I have had fun trying to restore that complexity by designing/revegetating using endemic plant species and watch the degraded areas flourish into ecosystems bursting with plant and animal species that are often rare or endangered in our area (e.g. Musk Ducks, Australian Shovellers, Pink-eared Ducks, Little Eagles, Swamp Harriers etc.). I was amazed at the vegetation that appeared in the mudflats and water during the last drought when our largest wetland was at its second lowest ever: Ranunculus sp., Schoenoplectus sp., Myriophyllum sp., Polygonum plebeium, Potamogeton crispus, Lachnagrostis filiformis, Crassula helmsii, Lythrum hyssopifolia, Persicaria lapathifolia etc. After the drought broke last year I loved seeing the native grasses and forbs nearly explode (e.g. Themeda triandra and Swainsona sp.). The edge of our largest wetland (which had ALL the vegetation removed in the ~80s by previous owners) is now thick with reeds after excluding grazing from the area; a huge contribution to an exceptional breeding season for our water birds. With time it will be lovely to see the larger trees and shrubs we have planted provide enhanced wetland/riparian habitat, ecological corridors and shaded areas for recreation.<br><br>I have strong memories as a child of spending time camping/swimming in a popular river spot with many casuarinas and my neighbour has memories of growing up along the Murray with the River Red Gums. We have incorporated those sentiments into how we have worked together to design and create an environment that provides us with ecologically resilient habitat, recreational opportunities as well as water security for our adjoining properties. This project is ongoing and a commitment for life; stewards of our land and water.
41	I hate that water is seen as a commercial commodity, would love to see more importance given to wetland health, environment, native animal habitat and traditional owners
42	I spend lots of time in the Murrumbidgee River corridor in the ACT walking, running, fishing, swimming and photographing. I enjoy the diversity and pleasantness riparian vegetation brings, and affects all the activities mentioned above. I am currently studying [….] in Environmental Science at [...]
43	I love hearing the little reed warblers calling when out canoeing. My son is also now just learning about how Bream need estuarine habitat for them to grow. But most importantly I love the fact that Lake Burley Griffin is able to be swum in because of the work that has gone into making flood catchment reservoirs with natural filters (vegetation)
44	The Gellibrand River in Victoria is only partially regulated and retains a natural floodplain. It has floodplain wetlands that span the full range of salinity from saltwater (next to estuary) to freshwater all along the river channel. There is a high diversity of native plants in all sections of the floodplain. Amazing saltmarsh veg near mouth which is highly coloured red, orange and gold in some seasons through to flowering floating plants in the freshwater section. Although grazed, each winter these floodplain wetlands form and plants emerge and grow. The whole water column is filled with a diversity of leaf shapes and forms, in an amazingly bright green. There are floating leaved plants too, and some with floating flowers. This includes one exotic, which has perfumed floating flowers and which adds aesthetically to the whole experience. There is a high diversity of frogs and invertebrates in these wetlands and the presence of one exotic amongst so many natives makes no difference to them. I would not like to see the vegetation or sediment disturbed to try and remove this plant, given that there has been no competitive exclusion and that disturbance may release nutrients that could cause a switch to phytoplankton-dominance. My point is - intact floodplains are precious, amazing places and that exotic floodplain plants do not always require remedial action, especially where such action could have significant adverse consequences.
45	I enjoy the variety of wetland and floodplain vegetation, and the seasonal and longer term changes.
46	As I have been fortunate enough to work in a variety of freshwater ecosystems, I have collected a myriad of memories from such environments. Some of my favorite include rivers and streams in the Pilbara, booming with life in the reeds and rushes along the banks. Providing habitat and shelter for fish and birds and frogs and breathtaking diversity of insects. Other memories from my personal life include camping trips to national parks where the highest density of diversity among the flora are found right near the riparian zone, including rare species such as orchids.
47	I was a child when Carp moved into the Namoi/Peel system and the River was heavily regulated by Keepit and Chaffey Dams.<br>It make me very sad to think that people today will not see big runs of Silver perch or witness Catfish nesting and courting in the shallows of a clear river.<br>I suggest fast track Biocontrol of European Carp, ensure stock have controlled access to streams and that all riparian zones are buffered with trees to keep water temperatures lower.
48	Water is life - healthy wetlands and floodplains support so many plants and animals, processes and diversity.<br>Joy of seeing response to flows and healthy wetlands and floodplains - dragonflies flitting, frogs & birds calling and roosting, sound of water<br>Healthy rivers, wetlands, floodplains provide personal and community benefits - mental and spiritual health.<br>Plants - Wavy marshwort, marsh club rush, flowering lignum, primrose, common reeds
49	Coorong. Renmark. Reeds, Potamogeton, ruppia

50	I know this survey is focusing upon the MDB, but I think most of my love and memories of wetlands comes from growing up in the NT. I used to visit wetland sites there (Fogg Dam, Kakadu and Litchfield National parks), and so I really value how the wetlands come alive after the monsoon. I think of lily pads, jacanas, egrets, spoonbills, and kingfishers and the sound of croaking frogs. Fogg Dam is probably one of my favourite spots as it is easier to visit than other sites and is a quintessential floodplain site. This is despite the modifications and building a "Dam wall" in attempt to grow rice a few decades ago.<br><br>Unfortunately the memories I have of the MDB are different. I have visited a few sites (ie. the Coorong, Renmark, Mannum, Walker Flat; all in SA), but I can see the degradation and intense agricultural use within the catchment. Although the Murray is beautiful driving along it, and partaking in recreational activities, I can't help but think of its poor ecological state that I know exists.
51	Paddling along the Murray riverbank viewing the plants, trees and reeds from the waters edge
52	There are so many wonderful wetlands, rivers and floodplains in NSW with so many values, it is difficult to choose a favourite. The sounds you hear in wetlands is one thing I find fascinating. The frogs croaking, birds calling and mozzies buzzing. When large groups of colonial waterbirds breed the sounds are amazing...if you can get over the smell! The different calls and rustling through the plants. I feel very privileged to have been one of the people to spend time in waterbird colonies to learn about their needs to successfully breed and watch chicks grow and learn new skills.
53	The transformation of floodplain landscapes can be brilliant. During the drought years 2017-2019 lignum communities on the Narran River floodplain were very stressed. Following good late summer and autumn rain which contributed to moderate flooding in the Narran system, the flow channels between the lignum stands were filled with Billy Buttons creating the look of rivers of gold flowing through the landscape.
54	Tall reeds (filters & habitat)
55	I love the water (stream flow) itself. Streams (especially in alpine regions) have something clean and peaceful about them.
56	Sitting next to two constructed ponds at my grandparents' home as a child. Swimming in the Murrumbidgee River at Kambah Pool and Pine Island. Finding private-ish spots to sit and relax along the Merri and Darrebin Creeks in Melbourne. Swimming in the upper Yarra. Taking my son to explore along the Cotter River, sharing my knowledge of which plants are native and which not. Spotting dragon flies. Helping with Waterwatch water quality testing and invertebrate monitoring for work. Building a frog bog in my back yard.<br><br>I don't seem to value particular species. I value any I know to be locally native to an area, or introduced but beneficial (theoretically - none come to mind). I didn't learn much about this as a child. I volunteered with a community-run natives nursery […] and joined Gardens for Wildlife through […..] and learned a bit there, and I am still in the process of growing my local knowledge in Canberra. My interest has never been specific to riverine and floodplain vegetation, but has encompassed all the ecosystems I have lived among.
57	Seeing vegetation respond to flooding (incl. e-flows) always brings me joy, although it does also illustrate how much has been lost.
58	I have many memories of playing, as a child, in and around creek beds and rivers, watching the animals and birds which fed or lived there, and the plants which grew each season. This was part of the simple joy of living. Even as a child I was aware of the observable far reaching effect of these water ways for plant, bird and animal life. I want future generations to benefit similarly from the simple and cost-free joy of living. Commercial demands on water are at a level which threatens flows, water health and support for biodiversity. The community at large needs to understand what they stand to lose in conceding, by inaction, to the wants of vested interests.
59	Just generally love walking along the riverside and enjoying the diversity.<br>Enjoy walking around wetlands and seeing how a variety of birds come and go.<br>Life would be greatly diminished without healthy rivers, wetlands and floodplains and the plants and animals that are present in these regions.
60	As a teacher I took students on activities into natural areas - including wetlands, such as Hattah - which, I believe, significantly added to the breadth of their education in various ways and lead to positive environmental outlooks. The same was true for our own family and contributed to an appreciation of natural areas & environmental values. I enjoy walking recreationally in small, (urban & more remote) wetland areas where wetland birds can be seen - egrets, spoonbills, herons, various waders, grebes, plovers, dotterels, avocets & others. As with all natural areas there is a tranquillity about such places and the sounds of nature.
61	Scientific research accessing waterways
62	The best stretches of river are where they still have the original or native regrowth native vegetation and these are few and far between.

63	Wading through beautiful clear waters in wetlands in the Tasmanian highlands where there are vast areas of different vegetation communities that occur in mosaic patterns. Absolutely stunning. Also finding a threatened native fish species spawning in shallow areas of submerged vegetation in this type of environment - very memorable and exciting at the time.
64	The loveliness of a native buttercup field in clear shallow floodwater under the filtered light of magnificent river red gums
65	Large expansive wetland complexes with abundant vegetation growth supporting large colonies of birds
66	all recreational places along the Murrumbidgee, Molonglo, Cotter River and others around Canberra, mostly for a swim or camping in summer, all species are of value (none in particular)
67	Australian rivers, even when intermittent, have vegetation that are different from the rest of the landscape. Animals such as birds and insects are also different because of this. What always strikes me is the diversity near water places and how it can change depending on how much water is present.
68	I have many memories of being a child and playing beside the river with my cousins in [….] in WA). We would use the plants and bushes beside the river to make little 'houses' with beds, kitchens and bathrooms. Bushes were shaped into walls, reeds were gathered to make bedding and seeds collected to fill the 'cupboards'. As we grew braver we would walk in the shallows of the river to small islands and create more little houses.<br>I would watch my brother and older cousin set traps nearby to catch marron and bring them home for everyone to eat.<br><br>Our family had a very close relationship to the land because of these experiences, and it lead to me becoming a professional in freshwater ecology. My understanding is that fostering a strong relationship with nature in young people is the best start to environmental education.
69	Ever since I was a child I would go down to the local river and hunt along the banks in the reeds for cool insects or small fish and frogs to keep in jars for a few weeks before returning them to the collection point. Water bodies such as rivers and wetlands have always had a significant part in both my education and the time I spent bonding with my family. Many of our family activities involved fishing or swimming.
70	Swimming in the rivers then settling in for a picnic on the banks under the trees (Cotter River, Murrumbidgee River).<br>Camping alongside the riverbank watching dragonflies, butterflies and other insects interacting with the vegetation.<br>Listening to the small birds in the shrubs.
71	Watching waterbirds nesting in wetland vegetation<br>Seeing small native fish and tadpoles in the shallows
72	Love swimming boating on river and being in a healthy eco system. If I didn't I would properly camp at the rubbish tip.. .look after it other wise it will be a dump. sorry a bit brief […]
73	Macquarie Marshes during a wet period was memorable for its breeding birds. Tropical wetlands in coastal north Qld and the NT (Kakadu) are exceptional all-year habitats for birds.
74	Rivers and wetlands are very peaceful places. I love seeing wetland and riverbank plants that are coming into flower. I like sitting very quietly on riverbanks or near wetlands to see and hear birds and frogs in amongst the reeds, and aquatic bugs swimming in amongst the aquatic plants. There are far too many favourite places for me to list. Where I live and everywhere I travel to I seek out rivers and wetlands to visit and spend time at, and plants are a key part of that experience.
75	All Creeks & waterholes dependent on floodwater downstream of Wyangala before the raising of damn wall
76	Wetlands and floodplains are remarkable systems, that are always changing with different years and water flows. The plant community is continually surprising with different flowers blooming. I partially love the change from dry to wet, when you come across a grassy patch one summer which burst into a wetland in spring, with stands of rushes surrounding the water, with running marsh flowers and Nardoo floating on the surface and those ephemeral patches packed with species of Mazus, Ranunculus and Crassula.
77	Freshwater mussels!
78	Some of my best memories of recreating in wetlands are strongly linked to vegetation conditions. Duck hunting in Heywood Lake after e-flows had filled it to over-spilling into Little Heywood. Inundating stands of knotweed gone to seed around the Lake perimeter. Grey teal and Black ducks were fat with crops full of knotweed seeds and some wheat from surrounding paddocks. Wood ducks were also plentiful feeding on the herb lawn as the lake level declined. The following year, grey teal were still abundant on the half full lake taking full advantage of the shallow dabbling habitat.<br>Kayaking through the flooded Hattah Lakes backwaters thick with red milfoil and flowering broad leaved pondweed (?), photographing ducks, spoonbills and white bellied sea eagles. Harvesting yabbies in Chalka Creek as their population boomed in response to the productive waters draining from the flooded vegetation filled floodplains.

79	Spoonbills, egrets and ducks foraging for small fish and invertebrates in dense swathes of Myriophyllum in Barmah-Millewa,
80	One strong experience I have had is up on the Barwon near Walgett - where the riparian zone was totally invaded by burrs. You couldn't get near the water even though there were substantial red gums there too. I was so struck by the transgression this represented to First Nations and their right and need to access waterways, and by the appalling investment in feral plant management in the Basin.

References

Bouman, T., L. Steg and H. A. L. Kiers (2018). "Measuring Values in Environmental Research: A Test of an Environmental Portrait Value Questionnaire." Frontiers in Psychology **9**.

Capon, S. J., L. E. Chambers, R. Mac Nally, R. J. Naiman, P. Davies, N. Marshall, J. Pittock, M. Reid, T. Capon, M. Douglas, J. Catford, D. S. Baldwin, M. Stewardson, J. Roberts, M. Parsons and S. E. Williams (2013). "Riparian Ecosystems in the 21st Century: Hotspots for Climate Change Adaptation?" Ecosystems **16**(3): 359-381.

de Casterle, B. D., K. De Vliegher, C. Gastmans and E. Mertens (2021). "Complex Qualitative Data Analysis: Lessons Learned From the Experiences With the Qualitative Analysis Guide of Leuven." Qualitative Health Research **31**(6): 1083-1093.

de Groot, R. S., M. A. Wilson and R. M. J. Boumans (2002). "A typology for the classification, description and valuation of ecosystem functions, goods and services." Ecological Economics **41**(3): 393-408.

Elo, S. and H. Kyngaes (2008). "The qualitative content analysis process." Journal of Advanced Nursing **62**(1): 107-115.

Ives, C. D. and D. Kendal (2014). "The role of social values in the management of ecological systems." Journal of Environmental Management **144**: 67-72.

Steg, L. and J. L. M. de Groot (2012). Environmental values. The Oxford handbook of environmental and conservation psychology. S. D. Clayton. New York, NY, Oxford University Press: 81-92.
